# Gene Editing and Genetic Control of Hemipteran Pests: Progress, Challenges and Perspectives

**DOI:** 10.3389/fbioe.2022.900785

**Published:** 2022-06-07

**Authors:** Inaiara D. Pacheco, Linda L. Walling, Peter W. Atkinson

**Affiliations:** ^1^ Department of Entomology, University of California, Riverside, Riverside, CA, United States; ^2^ Department of Botany & Plant Sciences, University of California, Riverside, Riverside, CA, United States; ^3^ Institute for Integrative Genome Biology, University of California, Riverside, Riverside, CA, United States

**Keywords:** Hemiptera, CRISPR/Cas9, whiteflies, gene editing, mutations, genetic control

## Abstract

The origin of the order Hemiptera can be traced to the late Permian Period more than 230 MYA, well before the origin of flowering plants 100 MY later in during the Cretaceous period. Hemipteran species consume their liquid diets using a sucking proboscis; for phytophagous hemipterans their mouthparts (stylets) are elegant structures that enable voracious feeding from plant xylem or phloem. This adaptation has resulted in some hemipteran species becoming globally significant pests of agriculture resulting in significant annual crop losses. Due to the reliance on chemical insecticides for the control of insect pests in agricultural settings, many hemipteran pests have evolved resistance to insecticides resulting in an urgent need to develop new, species-specific and environmentally friendly methods of pest control. The rapid advances in CRISPR/Cas9 technologies in model insects such as *Drosophila melanogaster*, *Tribolium castaneum*, *Bombyx mori,* and *Aedes aegypti* has spurred a new round of innovative genetic control strategies in the Diptera and Lepidoptera and an increased interest in assessing genetic control technologies for the Hemiptera. Genetic control approaches in the Hemiptera have, to date, been largely overlooked due to the problems of introducing genetic material into the germline of these insects. The high frequency of CRISPR-mediated mutagenesis in model insect species suggest that, if the delivery problem for Hemiptera could be solved, then gene editing in the Hemiptera might be quickly achieved. Significant advances in CRISPR/Cas9 editing have been realized in nine species of Hemiptera over the past 4 years. Here we review progress in the Hemiptera and discuss the challenges and opportunities for extending contemporary genetic control strategies into species in this agriculturally important insect order**r.**

## Introduction

Annually, insect pests decimate agriculture. The direct damage caused by pest feeding decreases the quality and yields of food, fiber, feed, and forage crops. Furthermore, the ability of some pests to vector phytopathogenic viruses and microbes further compromises agricultural productivity. Plant diseases and invasive pests cause an estimated $290 billion of loss to the global economy with losses caused by pests ranging from 20 to 40% of annual global crop production (FAO, 2019). While deployment of integrated pest management strategies limit losses, the current reliance on chemical insecticide applications and the emergence of insecticide-resistant pests has emphasized the pressing need for development of sustainable and environmentally sound practices for insect control, such as genetic control. Genetic-control strategies are species specific and designed to eradicate or replace insect pest populations, thereby providing an additional set of tools for effective integrative pest management. The principal targets of contemporary approaches for genetic control of pest insects have been dipteran and lepidopteran species. While these genetic-control strategies are advanced and, in some cases, almost ready for field deployment, significant hurdles remain (Hammond et al., 2021).

**FIGURE 1:**
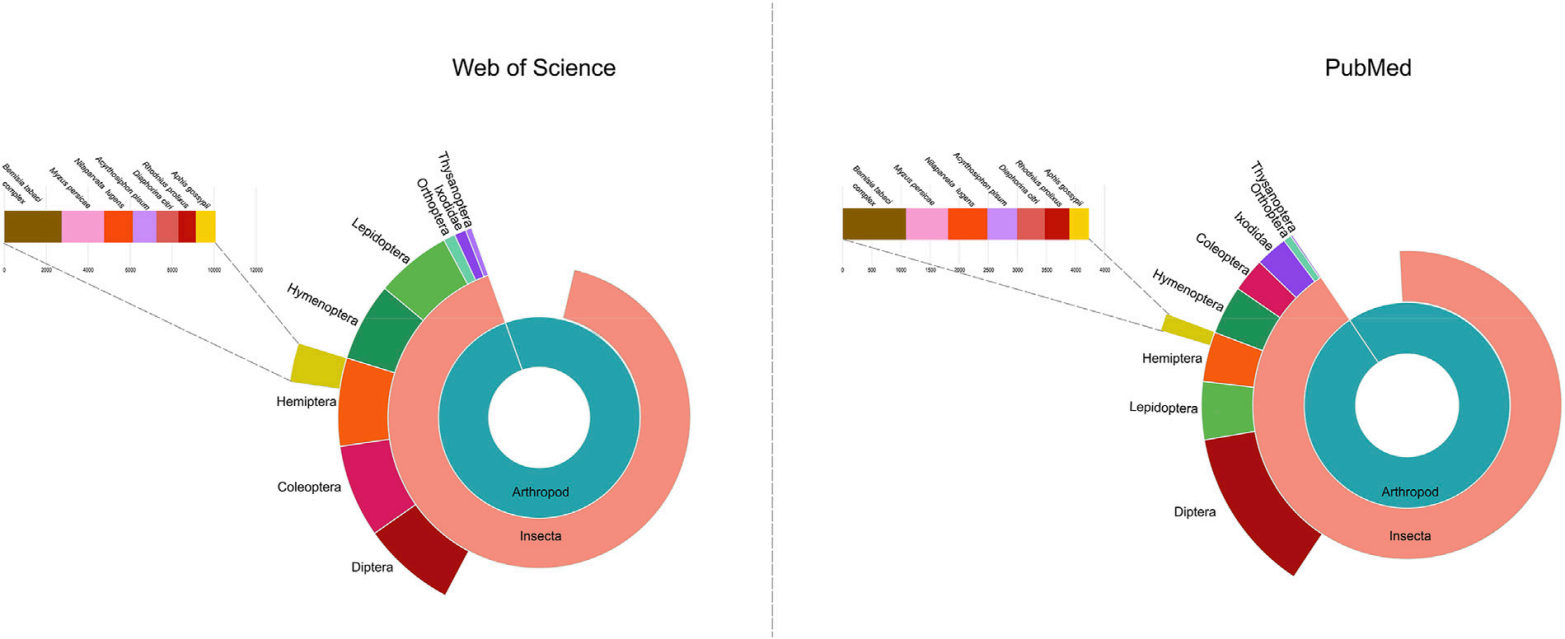
The number of publications on Hemiptera deposited in Web of Science and the National Library of Medicine (United States). The most numerous published species within Hemiptera are also shown. Data from 2012–2022

Most surprisingly, genetic control strategies for the Hemiptera are currently lacking, despite the importance of many hemipteran species as agricultural pests. Of particular importance to global agriculture are the sap-feeding species within the order Hemiptera. For example, the whitefly *Bemisia tabaci* is one of the top 100 insect pests world-wide with a broad host plant range (https://stateoftheworldsplants.org/2017/report/SOTWP_2017.pdf, Lowe et al., 2000. http://www.iucngisd.org/gisd/100_worst.php). The importance of phytophagous hemipteran pests is emphasized by trends in the literature. Of the 1,187 arthropod pests publications between 2012–2016 (Willis, 2017), four hemipteran species ranked in the top ten with: *B. tabaci* (#2), green peach aphid (*Myzus persicae*, #7), cotton aphid, (*Aphis gossypii*, #9), and brown planthopper (*Nilaparvata lugens*, #10). In addition, there was a significant increase in the number of publications focusing on insect pests of plants from 2007–2011 to 2012–2016; again, five of the top ten species were hemipteran. Our survey of publications identified in the National Library of Medicine at NIH and Web of Science identified over 128,367 and 135,944 publications on the Hemiptera, respectively, from 2012–2022 (Figure 1). In addition, many hemipteran species are invasive pests of agriculture. Members of the Hemiptera are the most abundant of non-native insect species in North America with near to 800 species of Hemiptera introduced since 1800 (Yamanaka et al., 2015; MacLachlan et al., 2021).

For both model and non-model insects, the enabling genetic tools derived from CRISPR/Cas9 gene-editing technologies have revolutionized insect biotechnology. CRISPR/Cas9 technologies enable efficient, cost-effective, precision mutagenesis that has been leveraged for improved and elegant strategies proposed for the genetic control of insect pests (Kyrou et al., 2018; Kandul et al., 2019; Hammond et al., 2021; Kandul et al., 2021; Li et al., 2021; Meccariello et al., 2021). Genetic-control strategies for all insect pests are completely dependent on the ability to genetically modify, through transgenesis or paratransgenesis, the target insect species and mass rearing of the target insect for deployment of these technologies in the field. Both have presented challenges in the Hemiptera. Genetic-control strategies for the mosquito *Anopheles gambiae* are being developed and cage tested (Hammond et al., 2021). Field release of genetically modified strains of *Aedes aegypti* that achieve population reduction has occurred in the Caribbean, Brazil, and the Florida Keys (Erickson, 2016; Schairer et al., 2021). Finally, a genetically modified strain of *Plutella xylostella* has been released in central New York to eliminate the local populations of this pest (Waltz, 2015).

The first genetic-control strategy to be deployed was the Sterile Insect Technique (SIT). SIT depends on the large-scale release of sterile males of a target species. Matings with females in the field are infertile, ultimately leading to target population decline (Bushland et al., 1955). SIT can control livestock and crop losses by eliminating these pest insects. For example, deposition of eggs within fruit by female fruit flies leads to the development and subsequent feeding of larvae causing massive damage and post-harvest yield loss; SIT reduces viable eggs, limits fruit damage and affords pest control. Other proposed genetic-control strategies primarily target the viability of females in the target species (Kyrou et al., 2018). As the number of females decline, there is a subsequent reduction in the overall population and prevention of pathogen vectoring. Pathogen vectoring is often associated with the activities of females; for example, female mosquitoes that transmit human pathogens when taking blood meals. SIT and other genetic-control strategies have been developed for insects that share a core of features that make them uniquely adapted to these genetic control mechanisms. These features include: exclusive sexual reproduction, males not being directly responsible for economic or medical impacts, cost-efficient mass-rearing, the ability to use antibiotics to regulate genetic control systems, and well-developed methods and tools for engineering insect genomes (Lance and McInnis, 2005; Robinson, 2005; Leftwich et al., 2014).

Unfortunately, target insect features that are essential for current SIT and genetic-control strategies in the Diptera and Lepidoptera are not always present in species of the Hemiptera. First and foremost, for the Hemiptera, both males and females can vector disease-causing pathogens and both cause significant feeding damage to plant hosts (Hogenhout et al., 2008). For example, *B. tabaci* males and females can acquire and transmit Tomato Yellow Leaf Curl Virus (Ning et al., 2015). For this reason, control methods that solely target males (i.e., irradiation for SIT) or females may not be applicable. Furthermore, little is known about the molecular basis of sex determination in Hemiptera. In the mosquito *An. gambiae*, the Mediterranean fruit fly *Ceratits capitata*, and the diamondback moth, *P. xylostella*, the mechanisms of sex determination have been determined at sufficient depth to enable the sex ratio to be modified (Fu et al., 2007; Jin et al., 2013; Kyrou et al., 2018; Meccariello et al., 2021). This is a critical step for control strategies dependent on the elimination of females. Unfortunately, this level of understanding is currently lacking for the Hemiptera with the exceptions of the brown planthopper (*N. lugens)*, the whitefly (*B. tabaci)*, and the kissing bug (*Rhodnius prolixus).* For these species, some of the key genes in sex determination have been identified and the differential splicing of their transcripts between the sexes determined but more research is needed before sex ratios can be altered as part of a genetic-control strategy (Xie et al., 2014; Guo et al., 2018; Zhuo et al., 2018; Wexler et al., 2019; Zhuo et al., 2021). In addition, some hemipteran species, such as whiteflies, are haplo-diploid and others lack Y chromosomes (Pal and Vicoso, 2015; Blackmon et al., 2017). These alternative genetic systems will, most likely, influence the design and efficiencies of genetic-control mechanisms (Champer et al., 2020; Li J. et al., 2020).

Second, cost-efficient mass rearing is not commonplace in the Hemiptera. Large-scale rearing has a large physical footprint making such initiatives space-, time- and cost-intensive. Third, phytophagous Hemiptera harbor obligate endosymbiotic bacteria, making the use of antibiotics to induce or repress transgene expression problematic. This negates the use of the popular tetracycline on/off bacterial system that has been used to regulate transgene expression in other insects (Thomas et al., 2000; Gong et al., 2005; Jin et al., 2013). Fourth, some Hemiptera have complex and environmentally regulated lifecycle features that make them challenging species for any technology that is dependent on sexual crosses. For example, many aphids reproduce parthenogenetically and the synchronous production of males and oviparous females is triggered by environmental cues (often only once per year) (Simon and Peccoud, 2018); this can be difficult to achieve in the laboratory, representing a significant technical challenge. In addition, other hemiptera have long generation times and significant annual diapause periods (Simon and Peccoud, 2018; Krugner et al., 2019). Fifth, the genetic toolbox required for routine insect biotechnology and the methods for introducing macromolecules (i.e., DNAs, RNAs, and proteins) are currently underdeveloped. Few constitutive and tissue-, cell- or stage-specific promoters and other regulatory elements have been isolated and shown to be active in Hemiptera. Finally, the methods for CRISPR/Cas gene editing are just emerging and standard methods for gene introduction has yet to be fully explored.

While many of the attributes that make Diptera and Lepidoptera amenable to SIT and other control strategies are not present in the Hemiptera, many of these constraints are not insurmountable. Current control strategies will need to be adapted or new strategies developed to enable the field of hemipteran genetic control. Within the past 4 years, there have been substantial advances in the field of hemipteran biotechnology. The enabling technology of CRISPR/Cas-mediated mutagenesis in the Hemiptera is emerging. The increasing numbers of annotated genome assemblies now provide essential components for the development of the genetic toolbox required for extending genetic control into hemipteran pests. Here we review the recent progress that has been achieved in extending CRISPR/Cas9 gene-editing technology into hemipteran species and offer perspectives on how these technologies may be further developed into genetic-control strategies for use in the field.

### Genome Assemblies of Hemiptera

**FIGURE 2:**
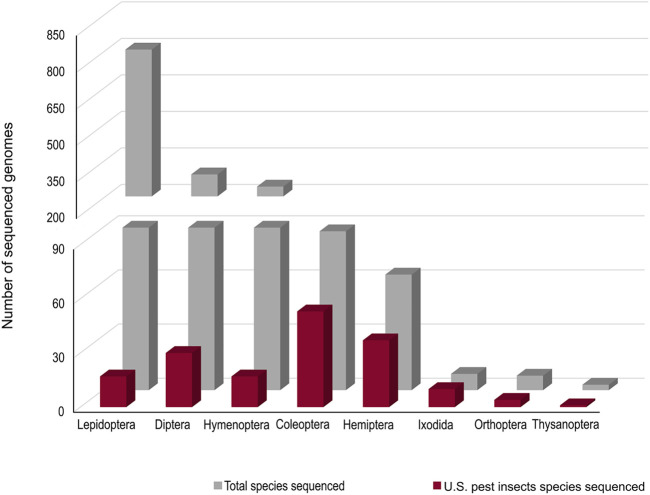
The number of genome projects for each species within each insect order based on depositions at the NCBI (all species) and at the i5K project (http://i5k.github.io/about). Data as at 2022.

**FIGURE 3:**
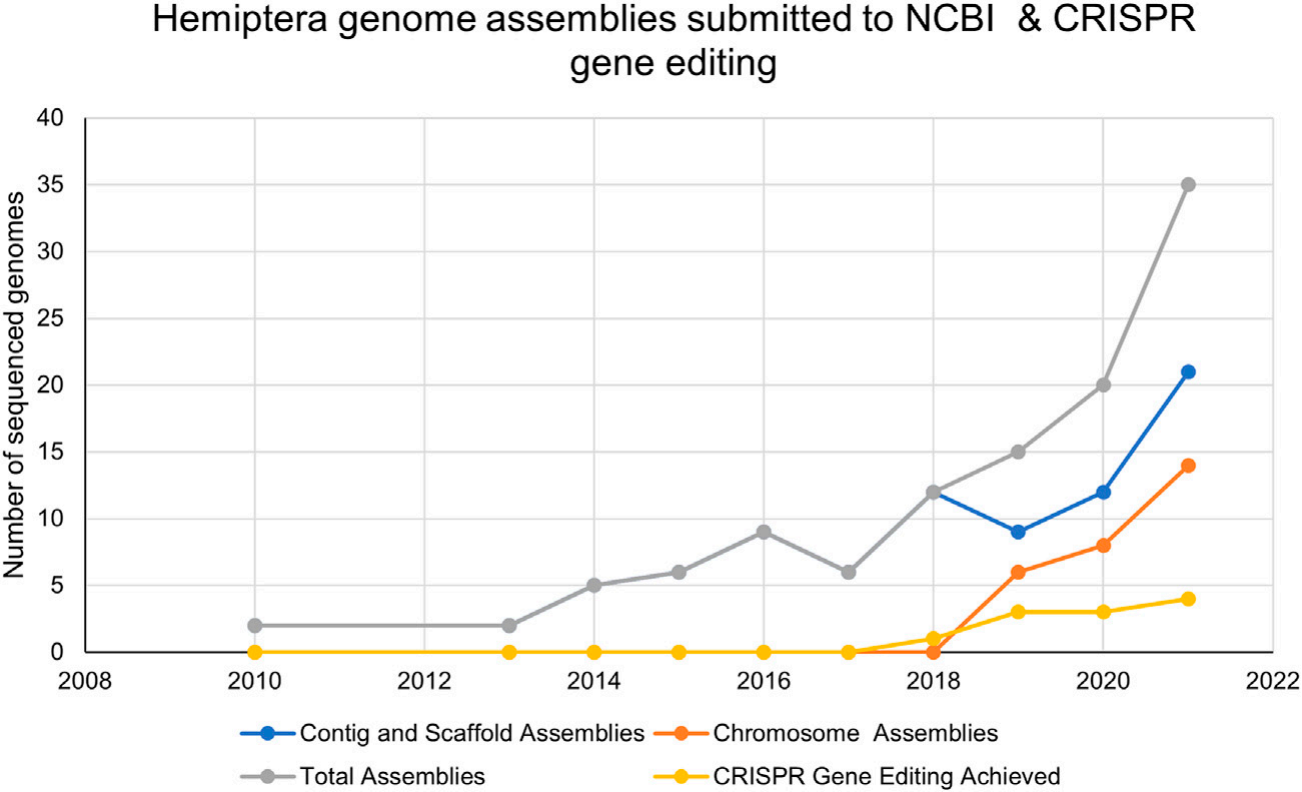
The number of Hemiptera genome assemblies at the NCBI and the number of publications reporting CRISPR/Cas9 mutagenesis in Hemiptera from 2010–2021.

**FIGURE 4:**
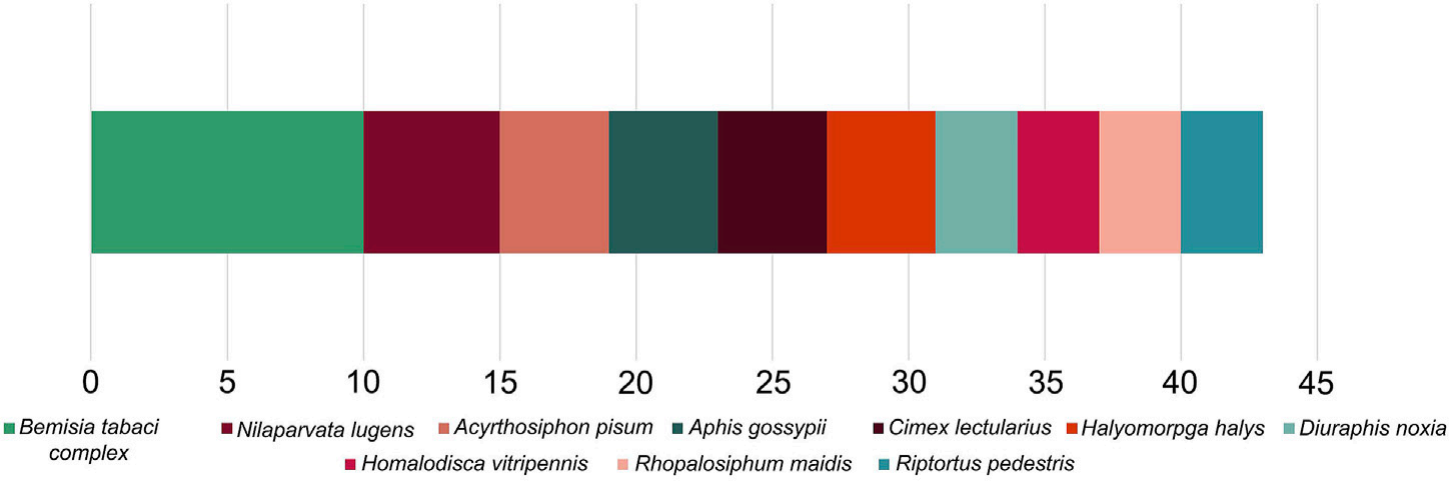
The ten most sequenced species (or species complex) within the Hemiptera with the number of submissions for each shown, based on NCBI data.

Accurate genomic information is critical for the development of genome-editing strategies. Well-annotated genomes facilitate the identification of target genes, the design of gene-specific sgRNAs (single-guide RNAs) and the identification of off-target sequences that could compromise the specificity of mutagenesis. Of the 26 insect orders, eight have genome projects. At present, there are 2,790 insect genomes accessible at NCBI (ncbi.nlm.nih.gov) and 114 are from the Hemiptera, representing 63 different species (Figure 2). The Hemiptera rank fifth, trailing the Lepidoptera, Diptera, Hymenoptera, and Coleoptera. When the genomes of insect pests of US agriculture are considered (i5k, 2022), the Coleoptera and Hemiptera predominate (Figure 2). Currently, a minute amount of the genomic diversity of the Hemiptera has been captured as more than 86,000 hemipteran species have been identified (ELO, 2022). The number of completed hemipteran genome projects has risen markedly since 2018, with chromosome-level genome assemblies first appearing in 2018 and increasing each year thereafter (Figure 3; Table 1). For the *B. tabaci* species complex, there are ten genomes available and, for nine other Hemiptera, there are three or more independent genomes (Figure 4, Supplementary Table S1). This provides insights into the sequence diversity associated with hemipteran species complexes and biotypes. All but one of these species (the bed bug, *Cimex lectularis*) are significant agricultural pests. *B. tabaci*, *N. lugens*, *Acyrthosiphon pisum (*pea aphid*)*, and *Homalodisca vitripennis* (glassy-winged sharpshooter) are discussed in more detail in this review, as their genomes have been leveraged for CRISPR/Cas9-mediated gene editing.

**TABLE 1:** Chromosomal assembly genome projects in Hemiptera deposited at NCBI.

Suborder	Family	Organism name	Number of Genes	Chromosome Number (including Sex Chromosome)	Sequenced Sex Chromosome (Sex Determination)	Mitochondrial Chromosome	References
Auchenorrhyncha	Delphacidae	*Laodelphax striatellus* (small brown planthopper)	16,412	15	X (X0)	YES	Noda and Tatewaki, (1990), Song and Liang, (2009), Ma et al. (2021)
		*Nilaparvata lugens* (Brown planthopper)	21,385	16	X,Y (XY)	YES	Song and Liang, (2009)
Heteroptera	Alydidae	*Riptortus pedestris* (bean bug)	21,562	6	X (X0)	YES	Hua et al. (2008), Kaur et al. (2012), Huang et al. (2021)
	Miridae	*Apolygus lucorum* (Mirid Bug)	20,353	17	-	YES	Liu et al. (2021)
		*Cyrtorhinus lividipennis*	14,644	13	X,Y (XY)	YES	Bai et al. (2022)
	Pentatomidae	*Aelia acuminata* (Bishop’s Mitre)	-	8	X,Y (XY)	YES	Crowley and Barclay (2021)
Sternorrhyncha	Aphalaridae	*Pachypsylla venusta* (hackberry petiole gall psyllid)	19,976	12	X (X0)	-	Yiyuan Li et al. (2020)
	Aphididae	*Acyrthosiphon pisum* (Pea aphid)	21,915	4	X (X0)	YES	Mandrioli et al. (2011), Li et al. (2019)
		*Aphis gossypii* (cotton aphid)	15,188–18,245	4	X (X0)	YES	Jaquiery et al. (2012), Zhang et al. (2022)
		*Eriosoma lanigerum* (woolly apple aphid)	28,186	6	X (X0)	YES	Biello et al. (2021)
		*Hormaphis cornu* (Witch-hazel cone gall aphid)	19,582	9	- (X0)	-	Korgaonkar et al. (2021)
		*Metopolophium dirhodum* (rose-grain aphid)	18,003	9	X (X0)	-	Zhu et al. (2022)
		*Rhopalosiphum maidis* (green corn aphid)	17,629	4	-(X0)	YES	Chen et al. (2019)
		*Sitobion miscanthi* (Indian grain aphid)	16,006	15	X (X0)	YES	Jiang et al. (2019)
	Pseudococcidae	*Phenacoccus solenopsis* (cotton mealybug)	11,880	5	-	-	Meizhen Li et al. (2020)

Of the 114 available hemipteran genomes (contigs, scaffolds, or chromosome-level assemblies) (Supplementary Table S1), only 25 contig/scaffold assemblies and 16 chromosome-level genomes are published (Table 1; Figure 3). A hemipteran genome explosion is beginning as 10 additional chromosome-level assemblies should be published soon (Supplementary Table S1). The high-confidence chromosome genome assemblies together with determination of chromosome number, the sex chromosomes, and gene numbers provide a foundation for the identification of gene targets and sgRNA design necessary for CRISPR/Cas gene editing (Table 1). As the number of completed and well-annotated hemipteran genome projects increases and genomes are re-sequenced to capture diversity within a species or species complex, the opportunities to conduct genetic research in these species will increase. It will be critical that these genome projects be of high quality in terms of the depth and breadth of coverage so that sgRNAs specific to unique target sites can be designed with confidence. Even with the existence of a reference genome for a given species, a laboratory, or local population of a species may need to be sequenced, at least across proposed target sites, in order to ensure that single-nucleotide polymorphisms (SNPs) do not confound sgRNA efficiency.

### Gene Delivery in Hemiptera

Two technologies have been used to assess hemipteran gene function: RNA interference (RNAi) and CRISPR/Cas9 gene editing. For RNAi, double-stranded RNAs (dsRNAs) are delivered to insects to transiently knock-down target gene expression yielding partial to full loss-of-function mutants. Delivery of dsRNAs to Hemiptera has been achieved by numerous techniques including: microinjection, artificial diets, petiole dips, and topical application. The successes and challenges associated with dsRNA strategies have been recently reviewed so will not be discussed here (Jain et al., 2020; Jain et al., 2021). In contrast, CRISPR/Cas-editing technologies can generate gene-specific mutations that are heritable and are often loss-of-function mutations (Jinek et al., 2013; Gratz et al., 2013; Yu et al., 2013; Bassett et al., 2013; Kistler et al., 2015; Meccariello et al., 2017; Wei et al., 2014; Jinek et al., 2012). Methods for efficient macromolecule delivery are essential to any CRISPR genome-editing system and the delivery strategy can be a substantial technological hurdle preventing deployment in target organisms. While temporally delayed by 3 to 5 years relative to the gene-editing advances in *D. melanogaster*, mosquitoes, the Lepidoptera, and the Coleoptera, CRISPR/Cas9-editing is now reported for nine species of Hemiptera with a total of 17 different genes being targeted (Tables 2–4) (Xue et al., 2018; Kotwica-Rolinska et al., 2019; Le Trionnaire et al., 2019; Zhao et al., 2019; Cagliari et al., 2020; Heu et al., 2020; Reding and Pick, 2020; Klobasa et al., 2021; Xue et al., 2021; Heu et al., 2022; Pacheco et al., 2022).

**TABLE 2:** The gene targets and efficiencies of CRISPR/Cas9-mediated mutagenesis in Hemiptera.

Species (common name)	Genome Available at NCBI?	Gene Target	Frequency of KO	Initial Technique of Detection	Frequency of G1 Gene Editing	Mutant G2 Generation Produced?	References
*Nilaparvata lugens* (Brown planthopper)	YES	*cinnabar*	0	Visual	48.8%	Not described	Xue et al. (2018)
		*white*	27.3%	Visual	3.2%	Not described	Xue et al. (2018)
		*Insulin receptor 1*	∼1–11.2% (plasmids) Not reported (Cas9 protein + sgRNA)	Deep sequencing of amplicons	9.1–35.7% (plasmids) 50–100% (Cas9 protein + sgRNA)	Not described	Zhao et al. (2019)
		*Insulin receptor 2*	Not described	Not described	Not described	Not described	Xue et al. (2021)
		*NICSAD*	9.5%	Sequencing	36.3% heterozygous	13.1% homozygous/53.3% heterozygous	Chen et al. (2021)
*Peregrinus maidis* (Corn planthopper)	NO	*white*	0.324	Visual	0	Not described	Klobasa et al. (2021)
*Homalodisca vitripennis* (Glassy-winged sharpshooter)	YES	*cinnabar*	58.9–66.7%	Visual	100%	100%	Pacheco et al. (2022)
		*white*	61.2–80.0%	Visual	100%	100%	
*Euschistus heros* (Neotropical stink bug)	YES	*yellow*	33.3%	Visual	0	Not described	Cagliari et al. (2020)
*Lygus hesperus* (Western tarnished plant bug)	NO	*cardinal*	100%	Visual	91.2%	A mix of wild-type and mutants	Heu et al. (2022)
		*cinnabar*	40–100%	Visual	77.9%	A mix of wild-type and mutants	
*Oncopeltus fasciatus* (Milkweed bug)	YES	*white*	14.0–92.5%	Visual	64.6% heterozygotes	No white homozygotes	Reding and Pick, (2020)
*Pyrrhocoris apterus* (Linden bug)	NO	*Cryptochrome 2*	35.7%	Heteroduplex assays	1.4–7.4% (1 sgRNA)	Not described	Kotwica-Rolinska et al. (2019)
		*timeless*	35.4%		4.2–66.7% (2)		
		*Period*	42.8%		0–61.5% (4)		
		*pigment dispersing factor*	83.3%		10.0–77.3% (2)		
		*TEFLamide*	43.3%		2.7–35.7% (2)		
*Acyrthosiphon pisum* (Pea aphid)	YES	*stylin-01*	66.7%,77.8%	PCR	35.3%	Not described	Le Trionnaire et al. (2019)
*Bemisia tabaci* (Whitefly)	YES	*white*	0.2–2.5%	Visual	Heterozygotes	21.4%	Heu et al. (2020)

Microinjection of preblastoderm embryos is the most common technique used for gene delivery in insects. Not surprisingly, this technology has dominated the hemipteran gene-editing experimental protocols. In eight hemipteran species, Cas9 protein, Cas9 mRNA, and crRNAs and tracRNAs, sgRNAs or plasmids expressing sgRNAs were directly microinjected into embryos. In most of these experiments, eggs were removed from the host plant and placed on a solid support platform. As *Lygus hesperus* eggs are usually deposited within the leaf and more difficult to excise, *L. hesperus* eggs were laid on parafilm gel packets for easy egg release for alignment for microinjections. The support varied from wet filter paper (*A. pisum*) to double-sided sticky tape on a glass slides (*N. lugens*, *Peregrinus maidis*, *Pyrrhocoris apterus*, *Euschistus heros*, and *L. hesperus*) (Table 4). In contrast, for *Oncopeltus fasciatus*, an agarose mold was constructed using *Drosophila* food-grade agarose. The mold held the eggs in position for sequential embryo microinjections (Table 4). A different approach was taken for *H. vitripennis*. Microinjection of these embryos occurred *in situ* on the leaf discs with penetration of the needle through the leaf *epidermis* and egg chorion into the embryo (Table 4). Finally, the approach for editing the whitefly, *B. tabaci*, was distinct as macromolecules were injected into the female abdomen (Table 4). This technique, called ReMOT Control (for Receptor-Mediated Ovary Transduction of Cargo) was developed as alternative to embryo microinjection (Chaverra-Rodriguez et al., 2018).

Not surprisingly, the size of insect eggs often influences the relative ease of embryo microinjection and the development of a high-efficiency gene-editing system. For some species, such as *H. vitripennis*, their larger (2.5 mm) embryos are exceptionally easy to inject (Pacheco et al., 2022). While the microinjection of the minute eggs of *B. tabaci* (∼0.1 mm in length) are more challenging but technically feasible using microinjection systems where the diagonal axis of the microinjector can be precisely controlled to avoid damage to the embryonic chorion and membranes. Precision injection parameters, as well as the choice of needle, can significantly enhance injected embryo survival to adulthood. Finally, hemipteran egg chorions can be very rigid, hard to remove and for some species hard to pierce. For this reason, Kotwica-Rolinska et al. (2019) soaked *P. apterus* eggs in water to soften the chorion prior to microinjection, which significantly decreased damage to the embryo and increased egg viability and hatch.

Both quartz and borosilicate glass needles have been used successfully to deliver macromolecules in the Hemiptera (Table 3). Beveled quartz needles were used to penetrate the chorions of *P. maidis*, *H. vitripennis* and *L. hesperus* and the cuticle and ovaries of *B. tabaci* adult females (Table 3). In contrast, borosilicate needles were used for microinjections of *O. fasciatus* and *P. apterus* embryos and glass microinjection capillaries tips and glass needles were used for embryo injections in *A. pisum* and *N. lugens,* respectively (Table 3).

**TABLE 3:** Microinjection protocols of CRISPR/Cas9-mediated mutagenesis in Hemiptera.

Species (common name)	Gene Target	Needle Type	Cas9 Protein (P) or mRNA (M) Concentration (ng/µL)	sgRNA or crRNA/trRNA Concentration (ng/µL)	Gene, Plasmid Concentration (ng/µL)	Dye	References
*Nilaparvata lugens* (Brown planthopper)	*cinnabar*	Glass	500 (M)	150 (1 sgRNA)	-	-	Xue et al. (2018)
	*white*	Glass	500 (M)	400 (1 sgRNA)	-	-	Xue et al. (2018)
	*Insulin receptor 1*	Not described	200 (P)	50 each separately (3 sgRNAs)	vasa-Cas9 (300)	0.2% phenol red	Zhao et al. (2019)
	*Insulin receptor 2*	Glass	500 (M)	Not described	-	-	Xue et al. (2021)
	*NICSAD*	Not described	200 (P)	50 (1 sgRNA)	U6a or U6b sgRNA (100)	0.2% phenol red	Chen et al. (2021)
*Peregrinus maidis* (Corn planthopper)	*white*	Quartz, beveled	500 (P)	400 each combined (3 sgRNAs)	-	20% phenol red	Klobasa et al. (2021)
*Homalodisca vitripennis* (Glassy-winged sharpshooter)	*White cinnabar*	Quartz, beveled	300 (P)	300 (1 sgRNA)	-	-	Pacheco et al. (2022), Al-Wahaibi and Morse (2009)
*Euschistus heros* (Neotropical stink bug)	*yellow*	Glass	300 (P)	300 (1 sgRNA)	-	-	Cagliari et al. (2020)
*Lygus hesperus* (Western tarnished plant bug)	*Cardinal cinnabar*	Quartz, beveled	300 (P)	150 each combined (2 sgRNAs)	-	-	Heu et al. (2022)
*Oncopeltus fasciatus* (Milkweed bug)	*white*	Borosilicate, tip opened with fine dissection scissors	300 (P)	80 each separately (3 sgRNAs)	-	-	Reding and Pick, (2020)
*Pyrrhocoris apterus* (Linden bug)	*Crypto-chrome 2*	Borosilicate, tip opened by gentle scratching with fine forceps	400 (M), 500 (P)	200, 400 (1 sgRNA)	-	-	Socha, (1993), Kotwica-Rolinska et al. (2019)
	*timeless*		500 (P)	200 (1 sgRNA)			
	*Period*		400 (M), 250, 500 (P)	200, 500 (1 sgRNA)			
	*pigment dispersing factor*		250, 400, 500 (P)	200 or 400 (1crRNA)/119 or 238 (1 trRNA), 200, 500 (1 sgRNA)			
	*TEFLamide*		400 (M), 250, 500 (P)	200 or 400 (1 crRNA)/119 or 238 (1 trRNA 200, 500 (1 sgRNA)			
*Acyrthosiphon pisum* (Pea aphid)	*stylin-01 (cuticular)*	Eppendorf Femtotips	333 (P)	40 each (4 sgRNAs)	-	-	Lin et al. (2014), Le Trionnaire et al. (2019)
*Bemisia tabaci* (Whitefly)	*white*	Quartz	BtKV-Cas9	250 of each combined (5 sgRNAs)	-	-	Heu et al. (2020)

As microinjection of developing pre-blastoderm embryos predominates the hemipteran gene-editing literature (Table 3), it is important to note the contrasting modes of development of the hemimetabolous Hemiptera relative to the holometabolous Diptera, Lepidoptera, Hymenoptera, and Coleoptera. Holometabolous insects are the more derived developmental state having evolved from hemimetabolous ancestors some 300 MYA (Labandeira and Phillips, 1996). As both Hemiptera and Holometaboloma embryos are microinjected before cellular blastoderm, these significant developmental differences would not be predicted to impact the efficiency of CRISPR/Cas9-mediated editing of the embryo’s germline cells. However, the differences in development could alter the age of the pre-blastoderm embryo and delivery site chosen for microinjection. In holometabolous species, some nuclei that contain CRISPR/Cas9-generated mutations will migrate towards the posterior pole and become germ-cell nuclei and mutations will be transmitted to future generations; while other nuclei containing mutations will become somatic cells and will only contribute to the mutant phenotype of the G0 generation embryo, larvae or adult (Mahowald, 2001; Dearden, 2006; Nakao et al., 2006; Schröder, 2006). In contrast, in the Hemiptera, germ cells are established later in embryonic development, with the likely exception of the pea aphid *Acyrthosiphon pisum* (Chang et al., 2006; Ewen-Campen et al., 2013). Therefore, microinjections, which result in nuclei that contain mutations generated during earlier stages of embryonic development, can be delivered to both the germline and somatic cells. For this reason, one would not expect to see any significant differences in the distribution of CRISPR/Cas9-generated mutations in the germline and somatic cells in G0 larvae and adults arising from microinjected embryos from the Hemiptera. At the present time, it is unclear if the fundamental differences between holometabolous and hemimetabolous insect embryonic development alters the distribution of alleles in germline *vs*. somatic cells; this awaits rigorous testing.

Regardless of the developmental program, embryo microinjection must be performed prior to pole cell formation and cellular blastoderm, so that injected macromolecules have immediate access to the nuclei before the formation of cell membranes. In holometabolous insects, such as *D. melanogaster*, mosquitoes (*Ae. aegypti*, *An. gambiae* and *Anopheles stephensi)* and the Mediterranean fruit fly (*C. capitata)*, the time to cellular blastoderm is short, typically less than 90 min (Morris et al., 1989; Foe et al., 1993; Loukeris et al., 1995; Catteruccia et al., 2000; Grossman et al., 2001). Short times to cellular blastoderm are not strictly associated with holometabolous insects. For example, cellular blastoderm initiates at 10 h for the lepidopteran *B. mori* and 8–9.5 h for the coleopteran *T. castaneum* (Takesue et al., 1980; Handel et al., 2000)*.* In contrast, the time to cellular blastoderm for the Hemiptera is not precisely established for all species used in gene-editing experiments. However, in the species examined to date, the duration of pre-cellular blastoderm is greater than 4 h and up to 20 h in length (Table 4).

**TABLE 4:** Post-injection protocols of CRISPR/Cas9-mediated mutagenesis in Hemiptera.

Species (common name)	Gene Target	Time to Cellular Blastoderm	Time of Embryo Microinjection (h after oviposition)	Support Platform	Post-injection Treatment	Days to Hatch	References
*Nilaparvata lugens* (Brown planthopper)	*Cinnabar white*	>4 h at 26°C	1–2 h	Glass slide to which aligned embryos were affixed by dissolved glue from double-sided sticky tape	Placed in Petri dishes and covered with moist filter paper rinsed in 20 ng/μL tebucanazole and 50 ng/μL kanamycin and placed in a walk-in chamber	9	Xue et al. (2018)
	*Insulin receptor 1*	>4 h at 26°C	2–3 h	Double-sided sticky tape, desiccated for 2–4 min, then covered with halocarbon 700 oil	Placed in a plastic slide box containing a moist paper towel. At 6–7 days developed embryos were transferred to Kimwipes and the mineral oil removed. Embryos were placed in rice sheaths	6–11	Zhao et al. (2019)
	*Insulin receptor 2*	>4 h at 26°C	1–2 h	Glass slide to which aligned embryos were affixed by dissolved glue from double-sided sticky tape	Placed in Petri dishes and covered with moist filter paper rinsed in 20 ng/μL tebucanazole and 50 ng/μL kanamycin and placed in a walk-in chamber	9	Xue et al. (2021)
	*NICSAD*	>4 h at 26°C	<2 h	Double-sided sticky tape, desiccated for 2–4 min, then covered with halocarbon 700 oil	Placed in a humidity chamber in a plant growth incubator	6–7	Chen et al. (2021)
*Peregrinus maidis* (Corn planthopper)	*white*	Not described	16 h	Double-sided sticky tape on a coverslip placed on 1% agar	Transfer coverslip with embryos to fresh 1% agar plate and place in humidity chamber	8	Klobasa et al. (2021)
*Homalodisca vitripennis* (Glassy-winged sharpshooter)	*Cinnabar white*	Undifferentiated stage lasts for ∼90 h	2 h	Sorghum leaf disc containing egg mass placed on 1% phytoagar	Leaf disc on phytogar placed in incubator	6–9	Al-Wahaibi and Morse (2009), Pacheco et al. (2022)
*Euschistus heros* (Neotropical stink bug)	yellow	Not described	1.25–2 h	Double-sided sticky tape on a glass slide covered with water and wrapped with plastic film	Transfer to wet filter paper soaked with 1% Nipagen in a Petri dish	7–8	Cagliari et al. (2020)
*Lygus hesperus* (Western tarnished plant bug)	*Cinnabar cardinal*	Not described	1 h	Double-sided sticky tape on a coverslip	Placed in 1% agarose Petri dishes	7–9	Heu et al. (2022)
*Oncopeltus fasciatus* (Milkweed bug)	*white*	20 h at 28°C	2–8 h	3% *Drosophila* food-grade agarose in a mold	Placed in a sterile Petri dish, physical removal of fungus growing on agar	7	Reding and Pick, (2020)
*Pyrrhocoris apterus* (Linden bug)	*Cryptochrome 2*	16–19 h at 25°C	0–2 h, 0–12 h	Double-sided sticky tape on a coverslip, covered with distilled water. Moisten embryos with water to soften chorion	Transferred to Petri dishes containing moist paper towels	7	Socha (1993), Kotwica-Rolinska et al. (2019)
	*timeless*		0–12 h				
	*Period*		2–4 h, 0–12 h				
	*pigment dispersing factor*		2–4 h, 0–12 h				
	*TEFLamide*		2–4 h, 0–12 h				
*Acyrthosiphon pisum* (Pea aphid)	*Stylin-01 (cuticular)*	>16 h	2 h	Embryos placed on wet filter paper on a glass slide	Embryos transferred to a Petri dish containing wet filter paper and placed in an incubator and subsequently transferred to plants	85	Lin et al. (2014), Le Trionnaire et al. (2019)
*Bemisia tabaci* (Whitefly)	*white*	Not applicable	Not applicable	Abdominal injections into adult females	Injected adult females transferred to a soybean leaflet in a Petri dish with a moist paper towels, then removed after 2 weeks and leaflet examined for gene-edited offspring	not applicable	Heu et al. (2020)

Tables 2–4 provide an overview of the parameters used gene-editing experiments discussed above in the Hemiptera and the resulting efficiencies of mutagenesis. The targets and associated phenotypes will be discussed below, but it is important to note that editing success has been achieved with different experimental strategies in different hemipteran species. Therefore, consensus parameters for optimal editing in the Hemiptera are difficult to define. For example, CRISPR/Cas9 mutants have been successfully achieved by microinjecting Cas9 protein complexed with sgRNAs in most hemipteran species tested and by microinjecting Cas9 mRNAs with sgRNAs in *N. lugens* (Table 3)*.* In addition, the concentrations of Cas9 protein varied markedly in these experiments ranging from 150 to 800 ng/μL. Furthermore, sgRNA concentrations varied five-fold (ranging from 80 to 400 ng/μL) and some protocols used sgRNAs complexed with Cas9, while others did not state whether they assembled the Cas9-sgRNA complexes prior to injection. Collectively, these diverse protocols indicate that variation in the quantities of the macromolecules required for editing are flexible, as there was no clear indication of an optimal concentration of either Cas9 protein, mRNA or sgRNAs across these nine species.

Other techniques have also been used to introduce nucleic acids into Hemiptera. For 15 different target genes, liposome-encapsulated dsRNAs were injected into *E. heros* second-instar nymphs (Castellanos et al., 2019). Gene silencing was observed for nine of these target genes and insect mortality exceeded 95% at 14 days post injection. While microinjection of liposomes into nymphs are an effective delivery vehicle for dsRNAs, oral delivery of liposome-encapsulated dsRNAs was less effective (Castellanos et al., 2019). Branched Amphiphilic Peptide Capsules (BAPCs) have been used as a delivery system for dsRNAs in the Hempitera and Lepidoptera. BAPCs are water-soluble nanoparticles composed of amino acids and were used to successfully deliver dsRNAs in liquid and solid diets to the pea aphid (*A. pisum)* and the red flour beetle (*T. castaneum*), respectively (Avila et al., 2018). In both insects, the gene target was the molecular chaperone BiP/GRP78, which plays a critical role in the endoplasmic reticulum’s unfolded-protein stress (UPR) response. Supplementation of the insect diets with BAPC-associated dsRNAs significantly enhanced gene silencing in both insects. In addition, dsRNAs for two additional targets were delivered to *T. castaneum* using BAPC particles: *Armet*, which is important in UPR, and *vermilion*, which is involved in the ommochrome biosynthetic pathway (Avila et al., 2018). Both liposome- and BAPC-mediated delivery have the potential to deliver the CRISPR/Cas9 editing machinery to Hemiptera embryos; to date, the use of these technologies have not been rigorously tested.

### Gene Editing in Hemiptera: Eye Pigmentation Mutants

As shown in Tables 2–4, CRISPR/Cas9-mediated gene editing has been achieved in nine hemipteran species in a relatively short period of time, as both genome projects and microinjection delivery protocols have become available. Overall, the mean frequencies of mutagenesis in the G0 generation varies from less than 1% in *B. tabaci* to as high as 100% in *L. hesperus* (Table 2). Eye-color pigmentation genes are the most common target genes as the ommochrome and pteridine pigment biosynthetic pathways are highly conserved across insects and mutations in these pigmentation genes provide an easily screened phenotypes (Vargas-Lowman et al., 2019). The utility of screening for eye-pigmentation phenotypes is relatively simple in insects with hemimetabolous development. For example, *H. vitripennis* and *L. hesperus* embryos with mutant eye color can be detected during the mid-late embryonic stages (Heu et al., 2022; Pacheco et al., 2022). Another advantage, especially important in the context of containment of transgenic insects, is that mutants can be identified prior to egg hatch, after which time nymphs are mobile. Plant hosts in appropriate containment cages can then be infested with nymphs with altered eye colors to determine the inheritance of the CRISPR-derived mutations.

Two eye-color genes *white (w)* and *cinnabar* (*cn*) have been used frequently for the development of CRISPR/Cas9-editing technologies in the Hemiptera (Tables 2–4). The most common gene target to date is the *w* gene that encodes an ABC transporter responsible for importing precursors for both the pteridine and ommochrome pathways into cells of the developing eye. *w* has been successfully edited in all six hemipteran species in which CRISPR technology has been deployed. G0 mutagenesis frequencies are measured by the percentage of mutant G0 nymphs/the total number of G0 nymphs that were recovered from microinjection experiments of embryos or adults (Table 2). The mutation efficiencies were highly variable with adult injections of *B. tabaci* ranging from 0.2–2.5% and embryo injections of *H. vitripennis*, *O. fasciatus* and *L. hesperus* reaching efficiencies as high as 80, 96, and 100%, respectively (Table 2). Intermediate mutagenesis efficiencies were observed in *N. lugens* (27.3%) and *P. maidis* (32.4%) (Table 2). It is also important to note that efficiencies can vary within a species. For example, for the most efficiently edited Hemiptera to date, efficiencies can vary 1.3- to 6.6-fold for *H. vitripennis* (61.2–80.0%) and *O. fasciatus* (14.0–92.5%), respectively (Table 2).

While these data speak to the versatility of the *w* gene as an efficient target for establishing CRISPR/Cas9 mutagenesis in the Hemiptera, the inheritance and viability of *w* mutant homozygotes differs dramatically between species studied to date. For example, no white-eyed *O. fasciatus* individuals were observed in the G1 generation. This is despite the fact that the mosaic-eyed individuals were identified in the G0 generation and mutant *w* alleles were verified by heteroduplex analysis in heterozygous individuals from the G1 generation at a frequency of 64.6% (Reding and Pick, 2020). Furthermore, no white-eyed *O. fasciatus* mutants were recovered in the G2 generation from matings of G1 heterozygotes, despite the prediction that they should be present in 25% of the offspring (Reding and Pick, 2020). The failure to obtain homozygous *w* individuals suggests that *w* mutant homozygotes are inviable. White-eyed G0 generation embryos of *P. maidis* were also observed, however, none of these embryos hatched. Furthermore, when wild-type G0 generation adults were mated, no white-eyed progeny were recovered in the G1 generation (Klobasa et al., 2021). These data, while not as comprehensive as those obtained from *O. fasciatus*, also suggest that mutations in the *P. maidis w* gene impact viability. For *L. hesperus*, injection of *w* dsRNAs into embryos caused embryo mortality prior to visible eye formation and for this reason CRISPR/Cas9 mutagenesis was not pursued (Heu et al., 2022). Viability problems arising from mutagenesis of the *w* gene have also been observed in *D. melanogaster* and the cotton bollworm *Helicoverpa armigera* (Borycz et al., 2008; Evans et al., 2008; Khan et al., 2017; Xiao et al., 2017; Ferreiro et al., 2018; Myers et al., 2021).

In contrast, inheritance of mutant *w* alleles and eye-color phenotypes were transmitted to the G1 generations in *N. lugens* (3.2%) and *H. vitripennis* (100%), and to the G2 generation in *H. vitripennis* (100%) (Xue et al., 2018; Pacheco et al., 2022). It is also noteworthy that *H. vitripennis w* mutants displayed a phenotypic pleiotropy in both wing, eye, and ocelli color, with red pigmentation of the wing veins and cells of the forewing being absent in *w* mutants (Pacheco et al., 2022). These experiments, in conjunction with the analysis of *H. vitripennis* cn mutants, revealed that the red pigmentation patterns on *H. vitripennis* wings are due to the red pteridine pigments, rather than red melanins as previously proposed (Timmons et al., 2011).

Finally, unlike the white-eyed or mosaic-eyed mutants of the Hemiptera described above, Heu et al. (2020) reported very different putative *w* mutant phenotypes in *B. tabaci*. They used an injection mix of five sgRNAs that were complexed with a fusion protein consisting of the Cas9 protein with a short *B. tabaci* vitellogenin-binding sequence. Injections were performed in the presence or absence of the endosome escape reagent saponin. While mutants were identified with the 0 and 4 μg/ml saponin injection mixes, higher saponin concentrations (8–16 μg/ml) were toxic. Orange-eyed 4^th^-instar nymphs and red-eyed G0 adults were detected. The G0 insects appeared to be genetic mosaics. Inheritance of mutant alleles were inferred from phenotypes from a cross of a G1 female and her red-eyed G0 father; a non-mendelian pattern of inheritance was observed (Heu et al., 2020).

Collectively these data suggest that mutations in *the w* locus of different Hemiptera have variable phenotypes and a variable fitness costs ranging from undetectable to severe. As such, although mutations in *w* have distinct, easy to screen phenotypes, *w* is not necessarily a “risk-free” target for the development of CRISPR/Cas9 technologies in the Hemiptera.

A second eye-pigment gene, *cn*, was successfully edited using the CRISPR/Cas9 machinery in *N. lugens*, *H. vitripennis* and *L. hesperus. Cn* encodes a kynurenine hydroxylase, which, like *w*, is involved in the ommochrome biosynthetic pathway. In *D. melanogaster, cn* mutants have bright red/orange eyes (Paton and Sullivan, 1978). Wild-type *H. vitripennis* has a complex eye-pigmentation patterning with marked brown striations over a cream colored background (Pacheco et al., 2022). *H. vitripennis cn* mutants were easily identified by mosaicism in the eyes of G0 embryos, late-stage nymphs and adults (Pacheco et al., 2022). The mutation frequencies for *cn* were robust ranging from 58.9–66.7% (Table 2). Following pair-matings of G0 mutant adults, *cn* eye phenotypes were recovered in 100% of G1 and G2 generation individuals and their mutant alleles were verified by DNA sequence analysis. The mosaic eye-color phenotype seen in G0 generation *H. vitripennis* individuals is consistent with *cn* being a cell autonomous genetic marker in this species, as it is in *Ae. aegypti* (Pacheco et al., 2022; Sethuraman and O'Brochta, 2005).

Similar to *H. vitripennis*, *L. hesperus cn* mutants embryos and G0 nymphs and adults had bright red eyes throughout their development (Heu et al., 2022). Based on the percentage of adults with mutant eye phenotypes, *cn* mutations were generated at high frequency ranging from 40 to 100%. These mutations were heritable and were transmitted into the G3 generation.

The phenotype of G0 generation *cn* mutants of *N. lugens* was distinctly different from *H. vitripennis* and *L. hesperus.* No *N. lugens* adults with the *cn* mutant eye-color were identified in the G0 generation. However, when G0 adults were pair mated, G1 *cn* mutant adults were identified at a low frequency (3.2%) based on their bright red/orange eye color and mutant alleles were verified by DNA sequencing (Table 2) (Xue et al., 2018). The lack of mosaic or eye-color phenotypes in the G0 insects suggests that the *N. lugens* *cn* is a non-autonomous marker. In *D. melangaster*, *cn* phenotypes are variable based on the *cn* allele ranging from non-autonomous (Beadle and Ephrussi, 1936) to autonomous (Paton and Sullivan, 1978). It was postulated that the variable *cn* phenotype could be due to insufficient amounts of 3-hydroxykyenureine in the circulating hemolymph of larvae leading to varied uptake of pigment into the eye during pupal development (Paton and Sullivan, 1978). This may occur in adults that are genetic chimeras having both wild-type and *cn* mutant proteins in their eye pigment cells. Therefore, the failure to detect the *cn* eye-color phenotypes in *N. lugens* G0 adults may indicate that *cn* could be non-autonomous in this species. Alternatively, the frequency of mutagenesis in the G0 insects may have been too low to detect mosaicism in the G0 *N. lugens* eyes due to the large amount of wild-type tissue present.

Neither Pacheco *et al.* (2022), Xue *et al.* (2018) nor Heu et al. (2022) reported fitness costs associated with mutations in *cn*. However, some mutant *cn* alleles have been associated with compromised viability in the mosquitoes *Ae. aegypti*, *An. stephensi* and *Culex quinquefascitus* (Pham et al., 2019; Bottino-Rojas et al., 2022). Gene-editing experiments in other Hemiptera are needed to resolve whether there are fitness costs associated with mutations in *cn* and whether *cn* is an autonomous or non-autonomous marker in the Hemiptera and insects from other orders.

The role of *cardinal (cd*), a second gene in ommochrome pathway, in *L. hesperus* eye color was examined by Heu et al. (2022). *Cardinal* encodes a haem peroxidase that converts 3-hydroxykynurenine into ommochromes. Like *cn*, *cd* editing occurred at high frequencies with 100% of the *surviving L. hesperus* adults being mutant. The impact of *cd* mutations were most visible in developing embryos and early instars, which showed red eyes. However, after the third instar, brown pigments gradually increased in the remaining nymphal stages and into adulthood. Although, *cd* mutant adults had redder eyes than wild-type insects, they were significantly darker than *cn* mutants. The authors suggest this is due to spontaneous oxidation of 3-hydroxykynurenine to form the brown xanthommatin (Li et al., 2017a; Figon and Casas, 2018; Zhuravlev et al., 2018).

### Gene Editing in Hemiptera: Disrupting Genes Associated With Cuticle Function, Peptide Perception and the Circadian Clock

In addition to creating mutants in hemipteran genes that control eye pigmentation, mutations in ten genes that influence a wide range of functions have been pursued. These genes include loci that impact: insect cuticle pigmentation (*CSAD* and *yellow*), a putative receptor for plant viruses (*stylin-01*), two insulin receptors (*InR-1, InR-2*), a neuropeptide of unknown function (*TEFLaminde*), and the regulation of the circadian clock (*cryptochrome, timeless, period*, and *pigment-dispersing factor*) (Tables 2–4).

Mutations in genes that control cuticle biogenesis and color have been used as phenotypic markers for assessing the success of RNAi and CRISPR/Cas9 editing strategies in the Hemiptera. Genes that influence the cuticle were successfully mutagenized using CRISPR/Cas9-mediated gene editing in *N. lugens, E. heros,* and *A. pisum* (Le Trionnaire et al., 2019; Cagliari et al., 2020; Chen et al., 2021). In *N. lugens,* Chen et al. (2021) studied mutants in the target gene *cysteine sulfinic acid decarboxylase* (*NlCSAD*), which influences dark melanin pigment accumulation. They compared the outcomes of silencing *NlCSAD* by RNAi versus generation of *NlCSAD* null mutations. Injection of dsRNAs into 3^rd^-instar nymphs reduced *NlCSAD* RNAs by 65% and increased melanin levels in the cuticle. To create homozygous null *NlCSAD* mutants, *NlCSAD* was successfully edited. No visible phenotype was observed in G0 embryos or developing nymphs due to the recessive nature of the *NlCSAD* mutant alleles. Therefore, sequencing of DNAs extracted from the discarded exuvia from hatched 5^th^-instar G0 nymphs was used to identify putative *NlCSAD* mutants. Mutations in the *NlCSAD* gene occurred at a frequency of 9.5% and seven mutant alleles were detected (Chen et al., 2021). This low frequency may explain the absence of phenotype.

To demonstrate inheritance, two mutant G0 generation females were outcrossed with wild-type males to generate G1 generation progeny, of which 36.3% displayed a darker cuticle than wild-type controls (Chen et al., 2021). Mutant alleles were verified using 5th-instar exuvia and G1 adults with identical alleles (a 4-bp deletion in *NlCSAD*) were mated to produce the G2 generation. The G2 offspring had a genotype ratio of wild-type:heterozygous:mutant of 1:2:0.5 and phenotypic ratio of 3:0.5 (wild-type:mutant). Dark pigmentation was well correlated with *NlCSAD* allele gene dosage and *NlCSAD* RNA levels. G2 generation insects homozygous for the *NlCSAD* null allele displayed a darker cuticle color than the *NlCSAD* heterozygotes or the *CSAD*-RNAi insects. While the biochemical basis for the underrepresentation of the recessive homozygotes was not determined, homozygous *NlCSAD* null insects had reduced female fecundity and egg hatch rates, suggesting a significant fitness cost. The Chen et al. (2021) experiments emphasize two critical points. First, pursuing CRISPR/Cas9-induced null mutants in genes with unknown phenotypic ramifications, such as *NlCSAD*, is feasible. Second, the use of 5th-instar exuvia to genotype individuals of each generation provided a simple, non-invasive mechanism to identify individuals in a timely manner to allow tactical genetic crosses to be performed and, thereby, allowing mutants to be identified in the absence of strong phenotypes.

Based on studies in a number of holometabolous insects and RNAi studies with the hemipteran twin-spotted assassin bug (*Platymeris bigattatus),* the *yellow* gene is thought to be involved in the synthesis of dark melanin pigments (Zhang et al., 2019). Cuticular pigmentation was studied in hemimetabolous *E. heros* using RNAi and by creating CRISPR/Cas9-edited mutants for two target genes - *tyrosine hydrolase* and *yellow* (Cagliari et al., 2020). dsRNA silencing of the *E. heros* tyrosine hydrolase showed a reduction in pigmentation, while silencing of *yellow* did not. For this reason, CRISPR/Cas9 was used to produce null alleles to resolve the role of *yellow* in stinkbug cuticle pigmentation. *E. heros* embryos were injected with Cas9 protein and a single *yellow* sgRNA (Table 4) (Cagliari et al., 2020). One G0 individual was recovered that had a 6-bp in-frame deletion in the *yellow* gene; this mutation did not disrupt the function of the *yellow* protein as this insect had wild-type cuticle pigmentation. To discover the role of *yellow* in hemimetabolous insects, *yellow* null alleles will need to be isolated in the future.

The protocols for CRISPR/Cas9 mutagenesis for the pea aphid (*A. pisum*) were established using the cuticular protein gene *stylin-01* as a target (Le Trionnaire et al., 2019)*.* Unlike *yellow* and *NlCSAD, stylin-01* does not have a role in cuticle pigmentation. Instead, s*tylin-01* may be receptor of noncirculative plant viruses. *Stylin-01* is specifically localized to the acrostyle of the maxillary stylets, an organ that is replete with receptors for plant viruses (Webster et al., 2018). Using *stylin-01* as a target of CRISPR/Cas9 mutagenesis, Le Trionnaire et al. (2019) developed an editing pipeline for *A. pisum,* which has a life cycle that is not well adapted to routine mutagenesis strategies. Like other aphids, progeny are primarily produced by parthenogenesis resulting in genetically identical clones (Simon and Peccoud, 2018). Sexual reproduction is triggered by a shortening of the photoperiod, thereby allowing for the production of males and viviparous females, which occurs once per year. Therefore, production of developmentally synchronized males and females for the production of fertilized eggs for microinjection technologies is a major limitation. A further challenge is the fact that fertilized eggs enter a 3-month, obligate diapause prior to the emergence of the sexually derived progeny (Simon and Peccoud, 2018).

These rather daunting life cycle challenges were tackled by Le Trionnaire et al. (2019) resulting in a 7-month protocol for CRISPR/Cas9 mutagenesis of *A. pisum.* Embryos were microinjected and viable, melanized eggs transferred to plant leaves for their obligate diapause period. Sequencing of pre-diapause embryos demonstrated that 70–80% of embryos had evidence of *stylin-01* gene mutagenesis. Egg hatch occurred, but at low rates (1–11%), and 17 G0 generation foundress aphids each gave rise to a clonal colony. Sequence analyses of the *stylin-01* gene in the progeny from these clonal colonies identified six G0 foundress aphids that contained multiple *stylin-01* mutant alleles. The zygotic inheritance of these alleles has yet to be demonstrated. An additional 7 months are needed to produce the developmentally synchronized males and oviparous females required for the crosses to assess allele inheritance and the role of *stylin-01* in non-circulative virus transmission in *A. pisum.*

Two insulin receptor genes (*NlInR1* and *NlInR2*) from *N. lugens* were targets of CRISPR/Cas9 mutagenesis (Tables 2–4) (Zhao et al., 2019; Xue et al., 2021). Previous studies showed that RNAi silencing of *NlInR1* and *NlInR2* controls wing polyphenism (short versus long wings, respectively), which is important for *N. lugens* dispersal (Xu et al., 2015). The null mutants of *NlInR1* and *NlInR2* generated by CRISPR/Cas9 mutagenesis indicated that the two classes of insulin receptors have distinct roles in *N. lugens* wing development. *NlInR1* and *NlInR2* mutants have some overlapping but also distinct phenotypes indicating their different roles in growth, development and adaptation to stress. For example, homozygous *NlInR1* mutants had an early embryonic lethal phenotype, while homozygous *NlInR2* mutants did not (Zhao et al., 2019; Xue et al., 2021). Heterozygous *NlInR1* mutants grew more slowly, had reduced body mass, shorter wings, and a longer lifespan relative to wild-type control insects. These phenotypes recapitulate the impact on *InR1* mutations on *D. melanogaster* growth and development and previous RNAi silencing of the *NlInR1* (Fernandez et al., 1995; Brogiolo et al., 2001; Tatar et al., 2001; Xu et al., 2015). The severity of the *NlInR1* mutant phenotypes was correlated with the domain of the *NlInR1* locus that was mutagenized; mutations in the leucine-rich repeat domain caused more severe developmental defects than those in the furin-like Cys-rich domain. Zhao et al. (2019) also examined the *InR1*-dependent transcriptome changes in the mutant versus wild-type *N. lugens* revealing that *NlInR1* regulates genes associated with numerous cellular processes including: insulin resistance, longevity, phototransduction, cellular metabolism, endocytosis, as well as protein biosynthesis and processing. Null mutants in *NlInR2* had different phenotypes than *NlInR1* mutants providing the first insights into the distinctive roles of this understudied InR2 receptor in insects (Xue et al., 2021). In contrast to *NlInR1* mutants, *NlInR2* mutants were not lethal, displayed accelerated cell division and cell proliferation in wings (which gave rise to long wings), had defective vein patterning, prolonged developmental time, and decreased fecundity.

In addition to the insights into *NlInR1 and NlInR2* functions, Zhao et al. (2019) established important tools and principles for CRISPR/Cas9 strategies in the Hemiptera. The authors used two distinct CRISPR/Cas9 mutagenesis strategies and both produced heritable mutations in *NlInR1*. First, they microinjected of Cas9 protein with two different *NlInR1* sgRNAs. In a second strategy, they microinjected custom-designed plasmids to express the two components of CRISPR/Cas9 machinery (Cas9 and sgRNAs) within the insect embryo. One plasmid expressed a *N. lugens* codon-optimized Cas9 protein using a 2.5-kb *N. lugens vasa* (*Nlvasa)* promoter, 5′-untranslated region (UTR) and 3′-UTR (*vasa:Cas9*). In addition, two *N. lugens U6* snRNA polymerase gene promoters (*U6a* and *U6b*) were used to drive the expression of *NlInR1* sgRNAs. These experiments represent the first time endogenous promoters were used for expressing the macromolecules for CRISPR/Cas9 editing in hemipteran embryos.

Zhao et al. (2019) also systematically analyzed the mutagenesis efficiencies using microinjected Cas9 protein and sgRNAs versus the *vasa:Cas9* and *U6:sgRNA* plasmids for gene editing. Microinjections using the *vasa:Cas9* plasmid instead of Cas9 protein increased the survival to adulthood by seven-fold perhaps due to the toxicity of the Cas9 protein (Zhao et al., 2019). Amplicon sequencing of G0 and G1 generations adults determined gene-editing frequencies. The frequency of inherited mutations in the G1 generation was higher in embryos microinjected with the Cas9 protein versus the *vasa:Cas9* plasmid. These data suggested that the immediate presence of Cas9 activity in embryos may be critical for efficient germline mutagenesis. In balancing the two findings, the authors concluded that the use of the plasmid DNAs as source of Cas9 was the optimal and more economical method for gene editing in *N. lugens*. The *vasa:Cas9* gene may be an important tool for increasing the efficiency of CRISPR-mediated editing in the future. If similar to other systems, integration of *vasa:Cas9* into the *N. lugens* genome by homology-directed repair or transposon mutagenesis will result in an increase in CRISPR/Cas9 editing to boost efficiencies of both knock-in and knock-out mutagenesis (Sebo et al., 2014).

A neuropeptide of unknown function (TEFLamide) and four genes associated with the circadian rhythm were targeted in *P. apterus* (Tables 2–4) (Kotwica-Rolinska et al., 2019). Microinjection of Cas9 proteins and sgRNAs into *P. apterus* embryos was performed*.* Putative gene edits in G0 and G1 generation individuals were initially identified by heteroduplex analysis and then confirmed by sequencing. The significance of these data is several-fold. First, an effective gene-editing protocol in *P. apterus* for five genes, whose mutations did not provide a clear morphological phenotypes to enable screening, was achieved. Second, the frequency of gene editing in G0 generation individuals, measured by heteroduplex assays, was extensive, ranging from 35.4–83.4%. The optimization of these relatively inexpensive heteroduplex assays to rapidly detect mutagenesis at the target sites served as their “phenotypic” screen. Third, the mutagenesis rates in embryos microinjected with *Cas9* mRNA vs. protein were compared. While G1 mutations were detected in G1 heterozygous individuals generated by *Cas9* protein injections, no mutants were recovered when *Cas9* mRNA was microinjected. However, this needs to be placed in context, as the total number of gene-editing events recovered was small (six gene edits from five G0 parents after 1,280 embryo injections) (Kotwica-Rolinska et al., 2019). In addition, these results contrasted those of Xue et al. (2018) in *N. lugens*, who detected *w* and cn mutants after microinjection of *Cas9* RNAs (97 gene edits from eight G0 parents and 1,064 microinjections); however, they did not directly compare editing efficiencies using microinjected *Cas9* protein vs. *Cas9* mRNA.

Kotwica-Rolinska et al. (2019) tested the efficacy of sgRNAs versus a two-component RNA system that used crRNAs and tracrRNAs using the *pigment-dispersing factor* (*pdf*) gene as the target. Both sgRNAs and crRNA/tracrRNAs generated G1 mutants. There was variation in replicate experiments and, given the small number of embryos injected, it is not yet possible to conclude if sgRNAs or the two-component system was more efficient. However, the reduced cost and availability of sgRNAs makes sgRNA use preferred (Kotwica-Rolinska et al., 2019).

Kotwica-Rolinska et al. (2019) also assessed if the age of injected embryos influenced gene-editing frequencies using Cas9 protein and *pdf* crRNA/tracrRNA. Cellular blastoderm occurs at 16–19 h following oviposition in *P. apterus* providing a large window of opportunity for microinjections (Socha, 1993). Interestingly, G1 mutants were detected at a frequency of 24.1% in embryos after an overnight oviposition (0-to-12 h-old embryos), while the frequency was 1.7% for embryos from a 2-to-4 h oviposition period (Kotwica-Rolinska et al., 2019). As these are the only data examining the timing of embryo microinjections in the Hemiptera and, despite the fact that a small number of mutants were generated per G0 adult, we may be able to conclude that, in those species with long pre-blastoderm periods, microinjection at later times may generate higher frequencies of gene editing. It would be interesting to further examine this using morphological makers that provide easier identification of gene-edited alleles.

Understanding the accuracy of CRISPR/Cas9 editing is critical for evaluating the biological impact of mutations generated by this system. In the Hemiptera, generation of off-target mutations has been examined in only two studies (Xue et al., 2018; Pacheco et al., 2022). Given the exceptionally high rates of CRISPR/Cas9-editing in *H. vitripennis* (Table 2), Pacheco et al. (2022) analyzed *w* and *cn* sgRNA specificity *in vivo*. Cas-OFFinder was used to identify potential off-target sites (Bae et al., 2014). Off-target candidates within transcribed regions of genes that had an exact match to the PAM site and all 7 bp in the seed region adjacent to the PAM site were candidate off-targets. The cutting frequency determination score was used to rank potential off-targets for their likelihood of being cleaved by the Cas9 protein (Doench et al., 2016). Finally, off-targets with nucleotide polymorphisms or bulges furthest away from the seed region were chosen for analysis, as they would be the most likely off-target sites. Four to five off-target sites for two *w* and one *cn* sgRNAs were chosen for analysis. Off-target mutagenesis was assessed in genomic DNA from four *w* G0 and six *cn* G0 females. Of the 11 amplicon libraries analyzed, the mean percentage of reads mapping to the off-target sites ranged from 0 to 0.95%; however, one *w* sgRNA had a mutation rate of 5.04%. These data indicated that off-target editing did not occur or occurred infrequently in G0 embryos. It is also important to note that as off-target mutations were assessed in G0 generation insects, their inheritance was not determined. The *H. vitripennis* off-target frequencies compared favorably to data from *An. gambiae* when off-target effects on a gene-drive strategy were assessed (Garrood et al., 2021).

Using the sgRNACas9 algorithm, Xue et al. (2018) identified a total of 54 and 100 putative off-target sites with 1–5 bp mismatches for the *N. lugens* *cn* and *w*, respectively. They determined mutations for four *cn* and three *w* off-target sites in three G1 *cn* and three G1 *w* mutants, respectively. Each of these off-target sites had five mismatched base pairs, with mismatches located at different proximities to the predicted Cas9 cut site. No evidence of off-target mutagenesis was detected. Collectively, these data from *H. vitripennis* and *N. lugens* indicate that off-target mutagenesis occurs infrequently at sites highly related to the sgRNA target site. The ability to accurately identify off-target sequences for sgRNAs is critical for successful and precise gene editing, both for unequivocally determining gene function and for genetic control strategies in which the genotype of a strain for release into the field needs to be known with certainty. These will require reference genome assemblies of high levels of accuracy to be available, as well as standardized criteria for objectively evaluating how to best identify off-target sites and how then to design mutagenesis experiments within them. Alternatively, or in addition, phenotypes from independently-generated mutant lines combined with several generations of out-crossing can also reduce or eliminate the effect of any off-target mutagenesis.

CRISPR/Cas9-mediated gene editing has not yet been used to generate gene insertions for gain-of-function mutants in Hemiptera. These “knock-in” mutations would be expected to occur at lower frequencies than the knock-out gene-editing frequencies reported based on studies in other insects (Table 2) (Gratz et al., 2014; Wang et al., 2017; Kandul et al., 2020). However, given the rapid extension of CRISPR/Cas9 technology into the Hemiptera, it is reasonable to expect that knock-in mutagenesis will soon be achieved.

### Somatic Assay Platforms: Hemiptera Cell Culture

Insect cell culture provide an opportunity to enhance and accelerate the experimental design of gene editing *in vivo* by testing parameters for efficient CRISPR/Cas9-mediated gene editing by identifying efficient sgRNAs and any potential off-target effects associated with the CRISPR/Cas9 mutagenesis. In addition, cell cultures allow the rapid testing of the ability of putative regulatory sequences (e.g, promoters and untranslated regions) to drive gene expression. The ability to use cell lines to optimize CRISPR/Cas9 mutagenesis in non-hemipteran insects is established. These studies have assessed: the ability of sgRNAs and Cas9 to cut target gene sequences, tissue-specific CRISPR, and optimization of Cas13 cutting (Meltzer et al., 2019; Viswanatha et al., 2019; Huynh et al., 2020; Trivedi et al., 2020).

Despite the fact that cell culture lines from 20 hemipteran species have been described and used primarily for use in plant-virus-vector interactions (Supplementary Table S2) (Boyapalle et al., 2007; Marutani-Hert et al., 2009; Jia et al., 2012; Wang et al., 2018), no CRISPR experiments have been performed in hemipteran cell cultures. However, an *H. vitripennis* cell culture has been used to assess the efficiency of RNAi after the delivery of *actin* dsRNAs via lipotransfection (Rosa et al., 2010). In addition, the activities of a small number of hemipteran promoters have been assessed in hemipteran, lepidopteran and dipteran cell culture lines (see Hemipteran promoters below) (Qian et al., 2016; Zhao et al., 2019).

Finally, insect cell cultures have been used to assess the activity of a small number of proteins in insecticide resistance. For example, *Sf9* cells from *S. frugiperda* were used to understand the roles of calcium-binding proteins and calmodulin in insecticide resistance in *B. tabaci* and G protein-coupled receptors in *H. halys* (Guo et al., 2019; Ahn et al., 2020; Guo et al., 2021). *D. melanogaster* cell lines were used to understand the impact of a mutation in the *Laodelphax striatellus* GABA receptor activity on resistance to insecticides (Nakao et al., 2011).

### Hemipteran Promoters

Germline and constitutive promoters are essential elements for any genetic toolbox used for the genetic manipulation of insects. Promoters can drive the expression of Cas9 *in vivo* to enhance CRISPR/Cas9 mutagenesis or fluorescent reporter genes to track transgene insertion events by CRISPR/Cas or transposons. To date, few hemipteran promoters have been isolated and characterized and no promoters from other insect orders have been tested in hemipteran cells culture or *in vivo*. Constitutive, germline-specific and tissue-specific promoters from target hemipteran species need be isolated and characterized. The hemipteran genetic toolbox requires RNA polymerase II and III promoters to drive the expression of mRNAs and small RNAs, respectively. Currently, the only tested endogenous hemipteran promoters are from planthopper species (Qian et al., 2016; Zhao et al., 2019).

*U6* promoters use RNA polymerase III to naturally direct the synthesis of small, highly abundant non-coding RNA transcripts in eukaryotes. *U6* promoters and their transcriptional terminators have been used to drive expression of sgRNA genes and small dsRNAs for gene-silencing strategies in planthoppers (Zhao et al., 2019). Two *U6* promoters, *U6a* (431 bp) and *U6b* (455 bp), were used in *N. lugens* CRISPR/Cas9 experiments*.* Microinjections using the *U6:sgRNA* plasmids showed that the *U6b* promoter outperformed the *U6a* promoter *in vivo* when the editing of the *N. lugens NlInR1* target gene was assessed (Zhao et al., 2019). At present a systematic evaluation of the benefits of using *U6:sgRNA* plasmids versus purified sgRNAs in embryo microinjection experiments has not been performed.

Constitutive polymerase II promoters from *N. lugens* and the green rice leafhopper (*Nephotettix cincticeps*) were identified, isolated, and tested (Qian et al., 2016). Three *N. lugens actin* genes (*Nl_act1-3*), two *N. cincticeps actin* genes (*Nc_act1-2*) and one *N. lugens α-tubulin* gene (*Nl_α-Tub*) were tested for their ability to drive the expression of a green fluorescent reporter protein (GFP) in insect cell culture lines. Each construct had ∼2-kb of promoter sequence and its associated 5′- and 3′-UTRs. Vectors with *promoter:GFP* constructs were transfected into S2 cells from *D. melanogaster*, Sf9 cells from *S. frugiperda* or BmN cells from *B. mori*. Only *Nl_act1-3, Nc-act1* and *Nl_α-Tub* were active (Qian et al., 2016). While *Nl_act3* and *Nc_act1* promoters were active all three insect cell lines, *Nl_α-Tub* was only active in S2 cells, albeit at very high levels (Qian et al., 2016). However, quite surprisingly, none of these promoters were active the *N. lugens* cell line tested (Qian et al., 2016).

These experiments show the value of using insect cell culture to systematically test promoter activity by following expression of a fluorescent marker. However, these data also serve as a cautionary note; while some constitutive promoters are active in multiple cell lines, others may have cell line-specific patterns of usage. Current cell lines cannot provide information about the tissue-specific usage of promoters; therefore, tissue specificity or constitutive expression is often inferred from conserved motifs within promoters, expression data, or data from orthologs of other insects in the literature. In this context, both the *Nl_act3* and *Nl_act2* group are most highly related to the muscle-specific actins from other insects (Qian et al., 2016). The activities of the planthopper *actin* and α-*tubulin* promoters *in vivo* await characterization by transient assays in microinjected embryos or after transgene integration into an insect genome.

Most germline-specific promoters that have been used extensively in insect biotechnology are derived from holometabolous insects (Adelman et al., 2007; Li et al., 2017b). There are fundamental differences in the mechanisms for germline formation in holometabolous and hemimetabolous insects, with the possible exception of *A. pisum*. Therefore, it is possible that a different set of germline promoters will be needed for the hemimetabolous Hemiptera. Based on sequence identity to the germline *vasa* of *D. melanogaster,* the *Nlvasa* gene was identified and its promoter was used to generate *NlInR1* and *NlInR2* mutants in *N. lugens* (Zhao et al., 2019; Xue et al., 2021). The ∼2-kb *vasa* promoter is the first RNA polymerase II-dependent putative tissue/cell-specific promoter has been used in the Hemiptera. Prior to the use of a plasmid expressing *vasa:Cas9* gene in embryo microinjection experiments, its ability to drive the expression of luciferase in *D. melanogaster* S2 cell cultures was demonstrated (Zhao et al., 2019).

While Zhao et al. (2019) demonstrated the activity of *vasa* in developing embryos and its ability to express sufficient quantities of Cas9 for gene editing in *N. lugens*, it is unclear if *vasa* is actually expressed in *N. lugens* germline cells. Given the differences in hemimetabolous and holometabolous germline development, it is possible that the *N. lugens vasa* will be expressed in a different manner than its *D. melanogaster* or mosquito orthologs. Germline establishment occurs by one of two mechanisms - maternal provision or zygotic induction (Extavour and Akam, 2003). Maternal provision is active in holometabolous insects, such as *D. melanogaster*. In this scenario, germplasm is generated during oogenesis and the germ cells form early in during embryogenesis at a specific site within the embryo. In contrast, zygotic induction requires the activation of the zygote genome before the germline is established and, accordingly, occurs later during embryonic development. Zygotic induction is the ancestral state of germline determination (Extavour and Akam, 2003).

The *Drosophila oskar* gene encodes a germ granule protein that is synthesized at the posterior pole of the oocyte. *Oskar* initiates germplasm assembly and germ cell determination; this gene appears to have been lost in the Hemiptera (Ephrussi and Lehmann, 1992; Lynch et al., 2011; Quan and Lynch, 2016; Blondel et al., 2021). For this reason, it is expected that there should be fundamental differences in the transcriptomes of embryos during oogenesis and early embryogenesis. This is supported from the analysis of *O. fasciatus* transcriptomes, which showed that 19 genes (orthologous to genes associated with germ cell formation in *D. melanogaster)* were expressed uniformly during these early embryonic stages (Ewen-Campen et al., 2013). This suggests that, at least in *O. fasciatus*, there is no maternal provision of the germplasm. In addition, the *O. fasciatus vasa* gene was not associated with germ cell formation but was required for spermatogenesis. In *N. lugens, Nlvasa:Cas9* and sgRNAs were active in the germ line and generated CRISPR/Cas9 mediated mutations that were inherited.

In addition, there are significant differences in expression programs of embryonic pair-rule genes. Pair-rule genes are expressed with different temporal and spatial patterns during embryogenesis in *D. melanogaster* and the hemipterans *O. fasciatus* and *Murgantia histrionica* (the harlequin bug) (Auman and Chipman, 2018; Reding et al., 2019; Hernandez et al., 2020). These surprising differences within the Hemiptera indicate that expression profiles of candidate germline-specific promoters from the Hemiptera should be characterized prior to use in CRISPR-based experiments that seek to specifically direct expression of transgenes to the male or female germline.

The rapid and successful development of CRISPR/Cas9-mediated knock-out mutagenesis in Hemiptera since 2018 increases the demand for tissue/cell-specific and constitutive promoters, which will be important for developing gain-of-function mutagenesis. Additional germline-specific promoters will need to be identified, as they are important for generating insect lines that express Cas9 in germline cells to enhance the frequency of gene insertion events *via* Cas9-mediated mutagenesis. Containment of Cas9 activity within the germline or early embryonic stages will be advantageous for reducing the number of off-target mutations, which could potentially confound any genetic strategy using CRISPR-mediated mutagenesis. Furthermore, identification of robust constitutive promoters that can drive fluorescent reporter gene expression *in vivo* will be a useful addition to the hemipteran genetic toolbox. Chimeric genes utilizing these promoters can be used as reporters to monitor gene integration *via* by CRISPR/Cas or transposon technologies. The *in vivo* activities of candidate promoters can be quickly screened in transient somatic assays in microinjected embryos, nymphs, or adults by their fluorescence. Alternatively, screens can occur in cell culture lines, with the caveat that not all “constitutive” promoters from the Hemiptera are active in all cell lines (Qian et al., 2016).

### Transposable Elements

Transposable elements are effective genetic tools for introducing and integrating exogenous DNA into the germline of insects. Until the extension of CRISPR/Cas9 knock-in technology using homology-directed repair into *D. melanogaster,* transposon-mediated gene integration was the only effective way generating gain-of-function mutants in insects (Gratz et al., 2014). Today, transposable elements are routinely used to integrate the CRISPR/Cas9 machinery, other transgenes, or reporter genes into insect genomes (Galizi et al., 2016; Li et al., 2017b; Meccariello et al., 2021). Insect lines that express Cas9 from their genomes are an important genetic resource used to increase the frequency of gain-of-function or loss-of-function mutations and are now utilized to enable the rapid development, testing and implementation of genetic control strategies for insect control. Insect transposable elements, such as *piggyBac* from the cabbage looper (*Trichoplusia ni)* and *Mos1* from *Drosophila mauritiana*, have wide host ranges and may be candidates for transposon-mediated mutagenesis in the Hemiptera. To date, transposon-mediated genetic transformation has yet to reported in the Hemiptera. However, the genomes of Hemiptera are replete with transposons that could enable this technology.

*A. pisum*, *O. fasciatus*, *H. halys*, *P. venusta*, *H. vitripennis*, and C. *lectularius* genomes have large numbers of *mariner-Tc1*, *Helitron*, *hAT*, and *piggyBac* transposons, with the *mariner-Tc1* superfamily being the most abundant (Peccoud et al., 2017). While an actively mobile transposon has not yet been identified in the Hemiptera, two full-length and potentially active *mariner*-like transposable elements (*Btmar1.1* and *Btmar2.1*) were recently identified in the *B. tabaci* genome (Zidi et al., 2021). Compared to *mariner* elements from other insect species, the *B. tabaci mariner* elements have longer terminal-inverted repeats. In the future, it will be interesting to understand if *Btmar1.1* and *Btmar2.1* can mobilize other *B. tabaci mariner* elements, if full-length *mariners* from other insect species can mobilize *B. tabaci mariner* elements, and finally if these mobile elements can function in other hemipteran species. The increasing numbers of well-annotated hemipteran genomes should facilitate these endeavors, as well as identifying if Hemiptera contain any novel active mobile elements from other transposable element superfamilies. This is quite feasible since an analysis of the abundance and diversity of transposable elements in 42 different arthropod species revealed that the hemipteran genomes have the greatest diversity of transposable-element superfamilies (Petersen et al., 2019). On average, hemipteran genomes contained a mean of 55.7 transposon superfamilies, compared to 48.5 superfamilies in the dipteran species (Petersen et al., 2019).

Insects with loss-of-function mutant alleles in eye-pigmentation genes may also have utility in development of efficient transposon mutagenesis strategies, as changes in eye color are easy to screen and can be detected in embryos, nymphs and adults (see section entitled Gene Editing in Hemiptera–Eye pigmentation mutants). This proposed strategy must exclude any Hemiptera in which null mutations in eye-color genes are accompanied by significant fitness costs or lethality. However, it is our experience that for some Hemiptera, such as *H. vitripennis*, *w* or *cn* mutant strains can be easily maintained for greater than six generations (Pacheco, Walling and Atkinson, unpublished). Such strains could serve as the parental genotypes for transposon-mediated gene insertion. Upon integration into the parental genome, transposons can deliver their cargo (a wild-type eye color gene under the control of constitutive promoter plus a gene of interest) to random sites in an hemipteran genome, thereby complementing the null *w* or *cn* mutation and restoring the wild-type eye color. This should provide a rapid and sensitive screen for transposable element insertion. As genomic safe-harbors (genome sites that allow high levels of transgene expression) are not yet characterized in the Hemiptera and most other insects, the use of eye pigmentation markers should rapidly advance the ability to generate and detect gain-of-function mutations. Alternatively, the transposable element’s cargo could be a fluorescent protein gene under the control of a constitutive or germline promoter; again providing a rapid and reliable screen for transgene insertion. Finally, as this technology develops, hemipteran lines that express a transposase gene under the control of an inducible promoter (i.e., a heat-shock promoter) would add additional ease to deploying transposon-mediated mutagenesis in the Hemiptera.

### Future Tools and Their Impact on Genetic Control Strategies

With the feasibility of editing hemipteran pests now established, we are entering a new phase of technology development focusing on the critical tools for deploying CRISPR/Cas9 editing. We will be able to interrogate developmental pathways in the Hemiptera and the biochemical and genetic pathways that underpin the interactions of hemipteran insects with their host plant species and the pathogens that they transmit. As we have seen in *N. lugens*, one of the most advanced hemipteran systems using CRISPR/Cas9 technology, new insights to insect gene function and evolution have already been revealed (Zhao et al., 2019; Xue et al., 2021). Furthermore, mutating *H. vitripennis’ w* and *cn* provided unanticipated insights into the origins of red pigments in forewings and the complex pigmentation patterns of *H. vitripennis* eyes. Finally, new genetic, chemical or behavioral solutions for the control of these insects will likely emerge.

To transition to development of genetic control mechanisms, there is now a pressing need for the development of efficient methods for creating gain-of-function mutants *via* gene integration. One limitation to deploying these technologies is the fact that genomic safe harbors for insect genomes are just beginning to emerge (Miyata et al., 2022). Genomic safe harbors are genome target sites that allow for the stable expression of transgenes without phenotypic consequences for the organism. For some Hemiptera, potential genomic safe harbors have already been identified such as *H. vitripennis’ w* and *cn* loci and *L. hesperus’ cd* and *cn* loci (Heu et al., 2022; Pacheco et al., 2022). For most Hemiptera, genome safe harbors will need to be identified. For this reason, transposon-mediated gene delivery is likely to come to the forefront as “random” sites of integration are used to identify genomic safe harbors that promote robust transgene expression. Subsequently, the presence or absence of detrimental impacts on hemipteran function can be assessed to identify the optimal sites for the integration of transgenes.

Once genome safe harbors for the Hemiptera are identified, the gain-of-function strategies for genetic control can be deployed leveraging the target-site gene integration methods afforded by CRISPR/Cas9 technology. Gene insertion cassettes can be introduced using CRISPR/Cas9-mediated homologous DNA repair or CRISPaint that uses nonhomologous end-joining (Gratz et al., 2014; Bosch et al., 2020). However, based on other insect systems where these technologies are established, these events are likely to be 10- to 100-fold less frequent than loss-of-function editing. For these reasons, strong tissue-specific and constitutive promoters will be needed drive robust visual reporters allowing for the development of time-savings, unambiguous phenotypic screens. In addition, the development of these technologies will also add a needed layer of rigor to current CRISPR/Cas9 genetic strategies that have been deployed in insects–the ability to prove unambiguously that a CRISPR/Cas9-derived mutation is the cause of a phenotype. Essential for this is the ability to complement a mutant and restore gene function; this is critically important given the fact that, while occurring at low to undetectable levels, off-target mutations can occur in CRISPR/Cas9-edited organisms. Alternatively, whole genome sequencing or extensive backcrossing will be needed to verify segregation of a phenotype with the mutation. Finally, another useful tool for genetic control will be the development of site-specific recombinase systems for the Hemiptera. These technologies will allow precise integration of genes into genomic safe harbors without risk of off-target site mutagenesis. In addition, these methods would allow the facile movement of gene cassettes in and out of the genomes of Hemiptera (Schetelig and Handler, 2013; Häcker et al., 2017; Schetelig et al., 2019).

With an improved set of genetic tools for the Hemiptera, contemporary genetic approaches, such as gene drive for population replacement or population elimination, may also now be pursued. However, a fundamental question exists - can gene drive strategies be developed and deployed in Hemiptera? As mentioned in the Introduction to this review, many Hemiptera lack the biological traits that easily enable genetics-based population control strategies, such as SIT. However, some Hemiptera have been considered excellent targets for genetic control. In assessing the potential use of CRISPR/Cas9 technologies for agricultural pest control, Scott et al. (2018) pointed to five potential target species: the new world screwworm fly (*Cochliomyia homnivorax*), the spotted wing *Drosophila* (*D. suzukii*), the diamondback moth (*P. xylostella*), the red flour beetle (*T. castanuem*), and the whitefly (*B. tabaci*). All of these species have high fecundity, short life cycles and are invasive species with global impacts on agriculture. We might argue that many hemipteran pests have these and other attributes that make these insects priorities for genetic control strategies.

The pros and cons of gene drive deployment as a genetic control in agriculture has been reviewed (Courtier-Orgogozo et al., 2017; Baltzegar et al., 2018; Legros et al., 2021). Furthermore, the dynamics of various gene-drive strategies for replacement or elimination of pest populations have modeled diploid pests with XY sex determination (Galizi et al., 2016; Hammond et al., 2016). However, some Hemiptera are haplodiploid and others lack a Y chromosome (XO) and these fundamental genetic differences may influence the efficacy of gene drive in these Hemiptera (Pal and Vicoso, 2015; Blackmon et al., 2017; Meccariello et al., 2021). Indeed, our models probing the efficiency of gene drive in *B. tabaci*, which has haplodiploid method of sex determination, indicate that if the alleles to be driven through a population have a small fitness cost or have a fitness advantage, the efficiency of the drive in haplodiploids is not appreciably different from drive systems in diploids (Li J. et al., 2020).

It may also be possible to develop gene-drive technologies for the Hemiptera that have longer life cycles, are not as fecund as *B. tabaci* and may be more problematic to rear. As noted by Legros et al. (2021), one advantage of agricultural targets is that their environment is managed, accessible and relatively small, unlike environments in native ecologies that are the geographic venues for gene edited-based mosquito gene-drive projects. An additional consideration to deployment of genetic control is the impact of a pest on its agricultural hosts in a specific region and stakeholder perceptions of gene-drive impacts. For example, for some host plant-pest interactions, feeding damage alone can suppress yields and require intervention; at these sites, genetic-control mechanisms to eradicate populations would be preferred. For other pests, losses are primarily associated with their ability to vector pathogens. In regions that are pathogen free, control strategies could focus on population replacement strategies with a benign gene-edited strain that cannot transmit the pathogen. Stakeholders may view population replacement with a benign strain a feasible strategy provided that gene-drive models are integrated with economic models and there is demonstrated cost savings to the stakeholder. A demonstrable and continual economic benefit for the stakeholder may mean that a program that takes several seasons to deploy and achieve its goal would be acceptable.

If a long-term view for control of a pest population can be embraced, low-threshold drives (release of modest number of gene-editing insects annually) could be deployed. Low-threshold drives maybe preferred since the release of large numbers of an edited strain has the potential to increase crop damage in the immediate term, which is not an economically desirable outcome. The use of low-threshold gene drives also reduces the need to have large mass-rearing facilities, which may not be feasible for some hemipteran pests. Finally, by using a private allele strategy in CRISPR/Cas-mediated gene drives, the control strategies can be contained and the drive’s impact can be limited to a specific target population (Willis and Burt, 2021). This specificity can be enabled by the precision editing controlled by Cas endonucleases and the choice of sgRNA sites that are present in a target population and absent non-target populations of the same species.

Based on strategies being developed for mosquitoes and the Mediterranean fruit fly, some of these control strategies may require manipulation of sex ratios (Kyrou et al., 2018; Meccariello et al., 2021). This will require a much greater understanding of the mechanism of sex determination in the Hemiptera. Mechanistically, the gene networks that control hemipteran sex determination must be revealed to assess if common or unique solutions to sex determination are used. To this end, orthologs of the *transformer*, *transformer2* and *doublesex* genes have been cloned and analyzed from *B. tabaci (tra, tra2, dsx*), *N. lugens* (*dsx*) and *R. prolixus* (*tra*, *dsx*); in addition, a novel gene that is a feminizing switch called *female determiner (Nlfmd*) has been identified as potential regulator of *dsx* in *N. lugens* (Xie et al., 2014; Guo et al., 2018; Zhuo et al., 2018; Wexler et al., 2019; Zhuo et al., 2021). CRISPR/Cas9-gene editing technology will provide a precise tool with which to accelerate our understanding of the mechanism of sex determination in Hemiptera and so provide opportunities for manipulating the sex ratio of target populations.

Identifying the reproductive biology and mating pattern of target species is mandatory for any successful gene-drive strategy. All gene drives depend on mating and fertilization between transgenic and wild-type individuals to bias the inheritance of a specific gene from one generation to the next (Dhole et al., 2020). Non-random mating can influence the spread of the drive, while inbreeding and multiple matings are of particular concern (Drury et al., 2017; Zentner and Wade, 2017; Champer et al., 2021).

Understanding a pest’s mating system is also essential to determine the dynamics of population growth for the target organism. For gene drives that promote male scarcity (Y-shredder) or polygynous populations (where males mate with multiple females) and only males are released, a much stronger bias in the sex ratio is required to achieve the same level of suppression than monogamous population strategies (Prowse et al., 2019). In addition, in sperm/male-killing strategies, male infertility can modulate female behavior by increasing female remating after ineffective matings (Kraaijeveld and Chapman, 2004; Charlat et al., 2007; Price et al., 2008; Iyengar and Reeve, 2010; Sutter et al., 2019; Sutter et al., 2021). Moreover, polyandrous mating systems, where females mate with multiple males, can limit the drive spread by reducing the probability that the egg will be fertilized by a drive-carrying sperm (Manser et al., 2020; Sutter et al., 2021). Multiple matings can also increase sperm competition (Iyengar and Reeve, 2010; Greenway et al., 2022). If the gene drive-carrying males have a reduction in sperm number and quality, sperm competition can hamper the reproductive ability of these animals and consequently suppress gene drive (Manser et al., 2017; Dhole et al., 2020; Manser et al., 2020; Price et al., 2020). These considerations are all pertinent to the development of future gene-drive strategies for the Hemiptera. Polygamy has been reported in several economically important Hemiptera such as stink bug, psyllid, mealybug, and glassy-winged sharpshooter (Mau and Mitchell, 1978; Seabra et al., 2013; Lubanga et al., 2018; Moura and Gonzaga, 2019; Silva et al., 2019; Cingolani et al., 2020; Gordon and Krugner, 2021).

Hemiptera present opportunities for genetic control in local, controlled, and managed environments. Gene-drive strategies must be adapted to the sex-determination mechanisms, mating propensities, life cycle features, and regional environmental conditions. Models to predict efficacy of a gene-drive strategy, as well as impacts on non-target organisms and the possible evolution of gene-drive immunity within the target population, will need to be developed. These models must also assess both short-term and long-term economic impacts to a region for stakeholders to embrace these longer-term solutions to hemipteran pest control.

Currently, there is indication of public support for gene drive applications for the control of insect pests of US agriculture (Jones et al., 2019). This study explored reaction to gene drive research in two insects of agriculture, the spotted wing *Drosophila* (*Drosophila suzukii*) and the hemipteran Asian citrus psyllid (*Diaphorina citri*). Public support was influenced by whether non-native or native species are targeted, whether the gene drive will be contained, and the entity conducting the experiments. The cost-effectiveness of the program and the speed of the gene-drive spread were of less concern than any impacts on human health and any adverse ecological impacts from removal of the target pest species (Jones et al., 2019). While other contemporary genetic technologies for inset pest control were not part of this survey, the authors hypothesized that similar public support would likely be extended to these, with the same qualifications (Jones et al., 2019). For these reasons, selected hemipteran pests may present an attractive platform for CRISPR/Cas-based genetic-control strategies. For example, a target pest species that was invasive, was confined to a managed environment in a specific geographical region isolated from related native species could present an opportunity for a controlled genetic-based elimination or replacement strategy that had economic parameters acceptable to the relevant industry and ecological parameters acceptable to other stakeholders.

## Conclusion

With current and emerging hemipteran genomes and the establishment of the CRISPR/Cas9 methods for creating mutations in target genes in hand, the hemipteran community is poised for rapid advances in the mutational analysis of these non-model insect species. Pipelines are now developed for nine hemipteran species. The protocols for the microinjection of Hemiptera embryos have now been developed and tuned to the unique biological characteristics of each insect. While protocols for some Hemiptera result in consistently high mutational frequencies, other protocols will need enhancements for larger scale deployment (Tables 2, 3). Collectively, the innovations that have been successfully deployed in the Hemiptera are remarkable. As reported in Tables 2 and 3, small advances like finding optimal rearing conditions to prevent *H. vitripennis* from entering its winter diapause (Pacheco et al., 2022), creation of molds to cradle fragile eggs for microinjection (*O. fasciatus*) (Reding and Pick, 2020), water soaking to soften tough chorions to facilitate microinjection and promote egg hatch (*P. apterus*) (Kotwica-Rolinska et al., 2019), use of gel-filled parafilm sachets for egg deposition (*L. hesperus*) (Heu et al., 2022), the development of cost effective methods to screen for allele inheritance non-invasively by extracting DNAs from nymph exuvia thereby demonstrating the ability to rapidly genotype individuals to identify edited G0 progeny without visible phenotypes (*N. lugens*) (Zhao et al., 2019), and exploration of the ReMOT system for the delivery of the CRISPR/CAS9 mutagenesis machinery (*B. tabaci*) (Heu et al., 2020), are examples of system innovations, some with high return. In addition, the persistence of hemipteran researchers to compress the daunting 12-month sexual mating cycle of the pea aphid to 7 months in controlled environments has allowed for the identification of CRISPR/Cas9-edited mutants (Le Trionnaire et al., 2019).

As CRISPR/Cas-mutational pipelines become more established, experiments will shift from the required technology development phase to addressing biological questions in the Hemiptera. This shift has begun for the brown planthopper, *N. lugens*. Pioneering advances in CRISPR/Cas9-editing of *N. lugens* began in 2018 and, by 2021, the analysis of CRISPR/Cas9-edited null mutants has provided critical and unequivocal new insights into roles of the pair of insulin receptors (*NlInR1* and *NlInR2*) (Zhao et al., 2019; Xue et al., 2021). As there is no ortholog of *InR2* in the model species *D. melanogaster*, the non-model insect *N. lugens* has already shed important light on *NlInR2* neofunctionalization (Xue et al., 2021).

**FIGURE 5:**
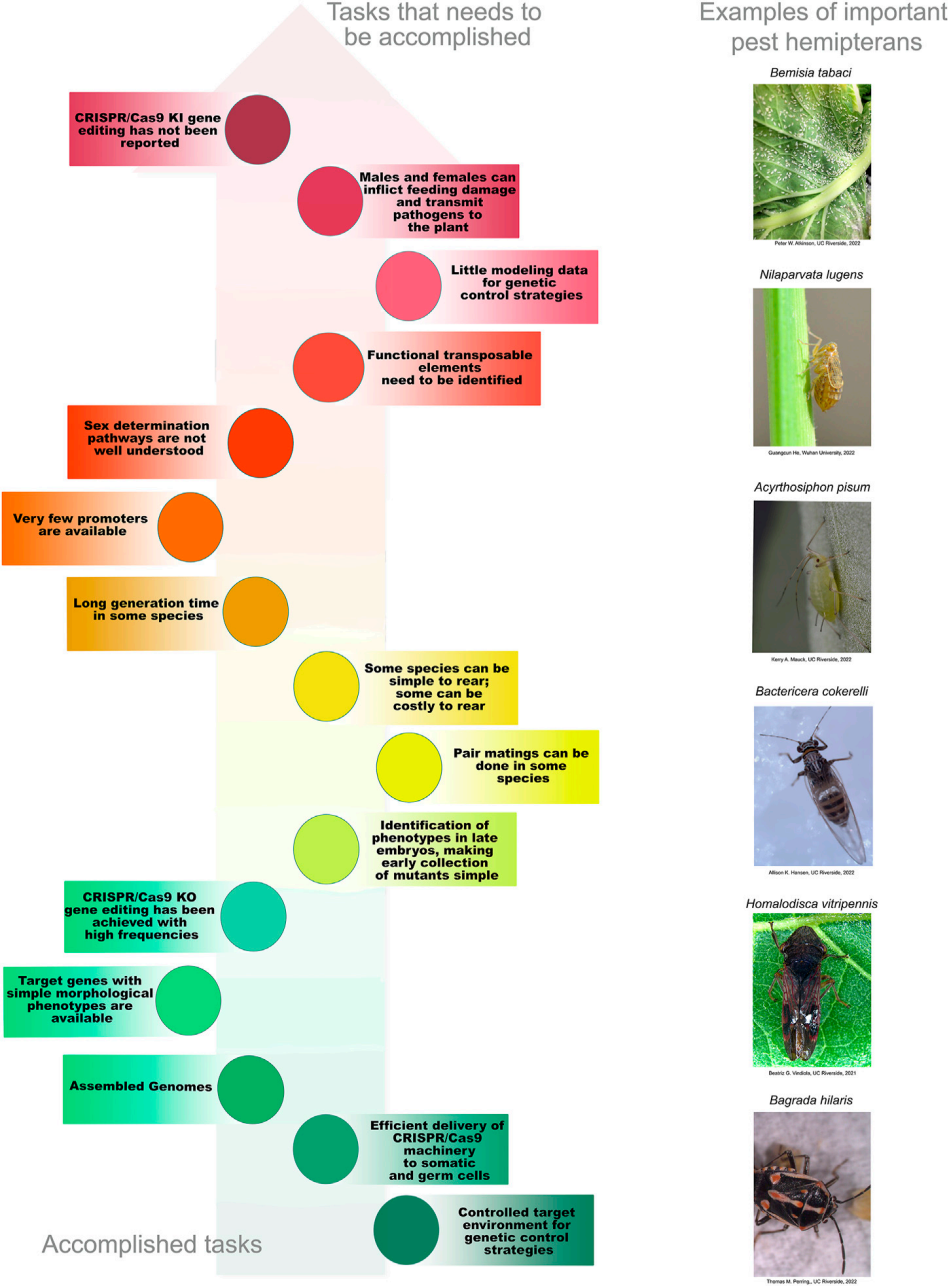
The progress made in establishing genetic technologies in the Hemiptera with green circles and panels depicting tasks that have been accomplished, pale green to tan circles and panels depicting relevant biological traits among Hemiptera that may affect our ability to efficiently extend genetic technologies to some species, and orange to dark red circles and panels depicting tasks that still need to be accomplished.

Progress in developing gene-editing technologies in Hemiptera and the steps remaining are shown in Figure 5. The next advances will depend on the accelerated development of the core tools needed for robust genetic analysis in the Hemiptera. We need a battery of promoters with different specificities to drive target genes, as well as reporter genes suited for robust screens in embryos and adults of non-model hemipterans. We need transposable elements and their associated transposases that function efficiently in the Hemiptera to carry and integrate their genetic cargo in the target insect genomes; this will enable the identification of genomic safe harbors for insertion of genes and gene cassettes needed to explore the biology, ecology and control of the Hemiptera. We need to develop of gain-of-function protocols for gene integration *via* CRISPR/Cas9-mediated homology directed repair, as well by nonhomologous end joining using CRISPaint protocols. This is likely to necessitate the development of hemipteran strains expressing Cas9 to enhance the frequency of these events. Finally, it is time to explore the advantages of other Cas endonucleases and the wide variety of CRISPR-mediated control strategies that can be deployed to interrogate gene regulatory programs in the Hemiptera.

## Supplementary Material

Supplementary Table S1 Genome projects of Hemiptera.

Supplementary Table S2 Cell culture lines of Hemiptera.
