# Supplementary material for: Gene Editing and Genetic Control of Hemipteran Pests: Progress, Challenges and Perspectives
Source: Front Bioeng Biotechnol. 2022 Jun 7;10:900785. doi: 10.3389/fbioe.2022.900785 (PMC9209771; doi:10.3389/fbioe.2022.900785)
Supplement: Supplementary file 1 [file Table1.docx]

**Supplementary table S1. Genome projects of Hemiptera.**

| **Suborder** | **Family** | **Organism Name** | **Organism Strain/Isolate** | **Assembly Accession** | **Annotation Name** | **Assembly Level** | **Reference** |
| --- | --- | --- | --- | --- | --- | --- | --- |
| Auchenorrhyncha | Aphrophoridae | Philaenus spumarius | PSPU_08 | GCA_018207615.1 | - | Scaffold | (Rodrigues et al., 2016) |
|  |  |  | PSP_Ac18 | GCA_002233535.1 | - | Scaffold | Unpublished |
| Auchenorrhyncha | Cicadellidae | Homalodisca vitripennis | AUS2020 | GCA_021130785.2 | - | Chromosome | Unpublished |
|  |  |  | HVIT.00 | GCA_000696855.2 | - | Scaffold | Unpublished |
|  |  |  | Tulare | GCA_019364655.1 | INSDC | Scaffold | (Ettinger et al., 2021) |
| Auchenorrhyncha | Cicadellidae | Magicicada septendecim | J4GVPIW7D5 | GCA_011326945.1 | - | Scaffold | (White and Pirro, 2021) |
| Auchenorrhyncha | Cicadellidae | Magicicada septendecula | SV565JUX3X | GCA_011763675.1 | - | Scaffold | Unpublished |
| Auchenorrhyncha | Cicadellidae | Matsumurasca onukii | IAE-20191127 | GCA_018831715.1 | - | Chromosome | Unpublished |
| Auchenorrhyncha | Delphacidae | Laodelphax striatellus | - | GCA_017141395.1 | - | Chromosome | (Ma et al., 2021) |
|  |  |  | Lst14 | GCA_003335185.2 | INSDC | Scaffold | Unpublished |
|  |  |  | SBPH | GCA_014465815.1 | - | Chromosome | Jiang et al., 2021, Laodelphax striatellus |
| Auchenorrhyncha | Delphacidae | Nilaparvata lugens | BPH | GCF_014356525.1 | NCBI | Chromosome | (Ma et al., 2021) |
|  |  |  | NLH13 | GCF_000757685.1/GCA_000757685.1 | NCBI | Scaffold | Xue et al., 2014 |
|  |  |  | BPH | GCA_014356525.1 | - | Chromosome | Unpublished |
|  |  |  | BPH2020 | GCA_015708395.1 | - | Chromosome | (Ye et al., 2021) |
| Auchenorrhyncha | Delphacidae | Sogatella furcifera |  | GCA_017141385.1 | - | Chromosome | Unpublished |
|  |  |  | WBPH | GCA_014356515.1 | - | Chromosome | (Ma et al., 2021) |
| Auchenorrhyncha | Fulgoridae | Zanna intricata | ZP7V8E2CI4 | GCA_010016005.2 | - | Scaffold | Unpublished |
| Heteroptera | Alydidae | Riptortus pedestris | Suzhou-2019 | GCA_019009955.1 | - | Chromosome | (Huang et al., 2021) |
|  |  |  | Nano-20200718 | GCA_020975785.1 | - | Scaffold | Unpublished |
|  |  |  | rp-2019 | GCA_021733305.1 | - | Scaffold | Unpublished |
| Heteroptera | Anthocoridae | Orius insidiosus | QHMPB2020 | GCA_014119065.1 | - | Contig | Unpublished |
| Heteroptera | Anthocoridae | Orius laevigatus | EB-2019 | GCA_018703685.1 | - | Scaffold | (Bailey et al., 2022) |
| Heteroptera | Belostomatidae | Lethocerus indicus | Cambodia-1 | GCA_019843655.1 | - | Scaffold | Unpublished |
| Heteroptera | Cimicidae | Cimex lectularius | Harlan/ CLEC.00 | GCF_000648675.2/GCF_000648675.1/GCA_000648675.3 | NCBI/INSDC | Scaffold | Unpublished |
|  |  |  | BBFD1 | GCA_001460545.1 | - | Scaffold | (Rosenfeld et al., 2016) |
| Heteroptera | Gerridae | Gerris buenoi | GBUE.00 | GCA_001010745.2 | - | Scaffold | Armisén et al., 2018 |
| Heteroptera | Miridae | Apolygus lucorum | 12Hb | GCA_009739505.2 | INSDC | Chromosome | Liu et al., 2021 |
| Heteroptera | Miridae | Cyrtorhinus lividipennis | HZCliv-2012 | GCA_019603395.1 | - | Chromosome | (Bai et al., 2022) |
|  |  | Dactylopius coccus | MexFresh/grana & fresh insects | GCA_000833685.1 | - | Contig | Unpublished |
| Heteroptera | Miridae | Nesidiocoris tenuis | - | GCA_902806785.1 | INSDC | Scaffold | Unpublished |
| Heteroptera | Miridae | Pachypeltis micranthus | Pm825 | GCA_020466155.1 | - | Chromosome | Unpublished |
| Heteroptera | Pentatomidae | Euschistus heros | 2730 | GCA_003667255.1 | - | Contig | Unpublished |
| Heteroptera | Pentatomidae | Halyomorpha halys | HHAL.00 | GCF_000696795.2/GCA_000696795.3/GCF_000696795.1/GCA_000696795.2 | NCBI/INSDC | Scaffold | (Sparks et al., 2020) |
| Heteroptera | Lygaeidae | Oncopeltus fasciatus | OFAS.00 | GCA_000696205.2 | - | Scaffold | Unpublished |
| Heteroptera | Pentatomidae | Piezodorus guildinii | RBSB-Aug2013 | GCA_000786065.1 | - | Scaffold | (Zucchi et al., 2019) |
| Heteroptera | Pentatomidae | Stiretrus anchorago | SMLB0N8UGS | GCA_010014745.1 | - | Scaffold | Unpublished |
|  |  |  | SMLB0N8UGS | GCA_913697995.1 | - | Contig | Unpublished |
| Heteroptera | Reduviidae | Rhodnius prolixus | - | GCA_000181055.3 | - | Scaffold | Unpublished |
| Heteroptera | Reduviidae | Triatoma infestans | FIOC_28 | GCA_011037195.1 | - | Scaffold | Unpublished |
| Sternorrhyncha | Aleyrodidae | Bemisia tabaci | MEAM1 | GCF_001854935.1/GCA_001854935.1 | NCBI | Scaffold | (Chen et al., 2016) |
|  |  |  | CAASIVF2012BtQ | GCA_003994315.1 | - | Scaffold | (Xie et al., 2017) |
|  |  |  | SSA1 | GCA_004919745.1 | - | Scaffold | (Chen et al., 2019b) |
|  |  |  | SSA1-SG1 Uganda F8 | GCA_902825415.1 | - | Scaffold | Unpublished |
|  |  |  | SSA1-SG1 Nigeria F8 | GCA_902825425.1 | - | Contig | Unpublished |
|  |  |  | Uganda Sweetpotato F5 | GCA_903994095.1 | - | Scaffold | Unpublished |
|  |  |  | - | GCA_903994105.1 | - | Contig | Unpublished |
|  |  |  | SSA3 Nigeria F8 | GCA_903994115.1 | - | Contig | Unpublished |
|  |  |  | SSA2 Nigeria F6 | GCA_903994125.1 | - | Contig | Unpublished |
| Sternorrhyncha | Aleyrodidae | Trialeurodes vaporariorum | IVF | GCA_011764245.1 | - | Scaffold | Unpublished |
|  |  |  | WFBEAN10G | GCA_009741425.1 | - | Scaffold | Unpublished |
| Sternorrhyncha | Aphididae | Acyrthosiphon pisum | AL4f | GCF_005508785.1/GCA_005508785.1 | NCBI | Chromosome | (Li et al., 2019) |
|  |  |  | LSR1 | GCF_000142985.2/GCA_000142985.2 | INSDC | Scaffold | Unpublished |
| Sternorrhyncha | Aphididae | Aelia acuminata | - | GCA_911387785.2 | - | Chromosome | Unpublished |
|  |  |  | - | GCA_911387705.1 | - | Scaffold | Unpublished |
| Sternorrhyncha | Aphididae | Aphis craccivora | 180601 | GCA_009835225.1 | INSDC | Scaffold | Unpublished |
| Sternorrhyncha | Aphididae | Aphis glycines | - | GCA_009761285.1 | INSDC | Scaffold | (Giordano et al., 2020) |
|  |  |  | OH | GCA_009928515.1 | - | Scaffold | Unpublished |
| Sternorrhyncha | Aphididae | Aphis gossypii | AGOS-L3 | GCF_004010815.1/GCA_004010815.1 | NCBI | Scaffold | (Quan et al., 2019) |
|  |  |  | Hap3 | GCA_020184165.1 | - | Chromosome | Unpublished |
|  |  |  | Hap1 | GCA_020184175.1 | - | Chromosome | (Zhang et al., 2022) |
| Sternorrhyncha | Aphididae | Aulacorthum solani | AS-CPRI-2016 | GCA_008528875.1 | - | Scaffold | (Tiwari et al., 2021) |
| Sternorrhyncha | Aphididae | Cinara cedri |  | GCA_902439185.1 | INSDC | Scaffold | Unpublished |
| Sternorrhyncha | Aphididae | Diuraphis noxia | RWA2 | GCF_001186385.1/GCA_001186385.1 | NCBI | Scaffold | (Nicholson et al., 2015) |
|  |  |  | - | GCA_001465515.1 | - | Contig | Unpublished |
| Sternorrhyncha | Aphididae | Eriosoma lanigerum | AA05 | GCA_013282895.1 | - | Chromosome | Unpublished |
| Sternorrhyncha | Aphididae | Hormaphis cornu | 80 | GCA_017140985.1 | - | Chromosome | (Korgaonkar et al., 2021) |
| Sternorrhyncha | Aphididae | Macrosiphum rosae | ROC1 | GCA_016617965.1 | - | Scaffold | Li et al., 2020 |
| Sternorrhyncha | Aphididae | Melanaphis sacchari | LSU | GCF_002803265.2/GCA_002803265.2 | NCBI | Scaffold | Unpublished |
| Sternorrhyncha | Aphididae | Metopolophium dirhodum | CAU | GCA_019925205.1 | - | Chromosome | (Zhu et al., 2022) |
| Sternorrhyncha | Aphididae | Myzus persicae | clone G006 | GCF_001856785.1/GCA_001856785.1 | NCBI | Scaffold | (Mathers et al., 2017) |
| Sternorrhyncha | Aphididae | Pachypsylla venusta | AUS-FW-20181119 | GCA_012654025.1 | - | Chromosome | Jiang et al., 2021 |
|  |  |  | PVEN.00 | GCA_000695645.2 | - | Scaffold | Unpublished |
| Sternorrhyncha | Aphididae | Pentalonia nigronervosa | sample_4 | GCA_014851325.1 | - | Scaffold | (Mathers et al., 2020) |
| Sternorrhyncha | Aphididae | Rhopalosiphum maidis | BTI-1 | GCF_003676215.2/GCA_003676215.3 | NCBI | Chromosome | (Chen et al., 2019a) |
|  |  |  | BTI-1 | GCF_003676215.1 | INSDC | Scaffold | Unpublished |
| Sternorrhyncha | Aphididae | Rhopalosiphum padi | XX-2018 | GCA_020882245.1 | - | Chromosome | (Chen et al., 2019a) |
|  |  |  | Rp-YL-2016 | GCA_019425515.1 | - | Scaffold | Unpublished |
| Sternorrhyncha | Aphididae | Sipha flava | LNK | GCF_003268045.1/GCA_003268045.1 | NCBI | Scaffold | Unpublished |
| Sternorrhyncha | Aphididae | Sitobion miscanthi | Langfang-1 | GCA_008086715.1 | - | Chromosome | (Jiang et al., 2019) |
| Sternorrhyncha | Aphididae | Sitobion avenae | Sa-YL-2016 | GCA_019425605.1 | - | Scaffold | Unpublished |
| Sternorrhyncha | Aphididae | Schizaphis graminum | BZ-2018 | GCA_020882235.1 | - | Chromosome | Unpublished |
|  |  |  | LNK | GCA_003264975.1 | - | Scaffold | Unpublished |
|  |  |  | Sg-YL-2016 | GCA_019425635.1 | - | Scaffold | Unpublished |
| Sternorrhyncha | Coccidae | Ericerus pela | colony RIRI-1 | GCA_011428145.1 | - | Scaffold | Unpublished |
|  |  |  | - | GCA_016591455.1 | - | Contig | (Yang et al., 2019) |
| Sternorrhyncha | Liviidae | Diaphorina citri | - | GCF_000475195.1/GCA_000475195.1 | NCBI | Scaffold | Unpublished |
| Sternorrhyncha | Pseudococcidae | Ferrisia virgata | - | GCA_900060175.1 | - | Scaffold | Unpublished |
| Sternorrhyncha | Pseudococcidae | Hypogeococcus pungens | - | GCA_018107765.1 | - | Scaffold | Unpublished |
| Sternorrhyncha | Pseudococcidae | Maconellicoccus hirsutus | vbl2512 | GCA_003261595.1 | - | Contig | Unpublished |
|  |  |  | - | GCA_900064465.1 | - | Scaffold | Unpublished |
| Sternorrhyncha | Pseudococcidae | Paracoccus marginatus | - | GCA_900065295.1 | - | Scaffold | Unpublished |
| Sternorrhyncha | Pseudococcidae | Phenacoccus solenopsis | jinhua | GCA_009761765.1 | - | Chromosome | Unpublished |
| Sternorrhyncha | Pseudococcidae | Pseudococcus longispinus | - | GCA_900064475.1 | - | Scaffold | Unpublished |
| Sternorrhyncha | Pseudococcidae | Trionymus perrisii | - | GCA_900050545.1 | - | Scaffold | Unpublished |
| Sternorrhyncha | Pemphigidae | Schlechtendalia chinensis | SC2021 | GCA_019022885.1 | - | Chromosome | Unpublished |
| Sternorrhyncha | Pemphigidae | Tetraneura ulmi | DLS19ref | GCA_021307375.1 | - | Scaffold | Unpublished |
| Sternorrhyncha | Veliidae | Microvelia longipes | CP-2014 | GCA_018340805.1 | - | Scaffold | Toubiana et al., 2021 |

References

BAI, Y., SHI, Z., ZHOU, W., WANG, G., SHI, X., HE, K., LI, F. & ZHU, Z. R. 2022. Chromosome-level genome assembly of the mirid predator Cyrtorhinus lividipennis Reuter (Hemiptera: Miridae), an important natural enemy in the rice ecosystem. *Mol Ecol Resour,* 22**,** 1086-1099.

BAILEY, E., FIELD, L., RAWLINGS, C., KING, R., MOHAREB, F., PAK, K. H., HUGHES, D., WILLIAMSON, M., GANKO, E., BUER, B. & NAUEN, R. 2022. A scaffold-level genome assembly of a minute pirate bug, Orius laevigatus (Hemiptera: Anthocoridae), and a comparative analysis of insecticide resistance-related gene families with hemipteran crop pests. *BMC Genomics,* 23**,** 45.

CHEN, W., HASEGAWA, D. K., KAUR, N., KLIOT, A., PINHEIRO, P. V., LUAN, J., STENSMYR, M. C., ZHENG, Y., LIU, W., SUN, H., XU, Y., LUO, Y., KRUSE, A., YANG, X., KONTSEDALOV, S., LEBEDEV, G., FISHER, T. W., NELSON, D. R., HUNTER, W. B., BROWN, J. K., JANDER, G., CILIA, M., DOUGLAS, A. E., GHANIM, M., SIMMONS, A. M., WINTERMANTEL, W. M., LING, K. S. & FEI, Z. 2016. The draft genome of whitefly Bemisia tabaci MEAM1, a global crop pest, provides novel insights into virus transmission, host adaptation, and insecticide resistance. *BMC Biol,* 14**,** 110.

CHEN, W., SHAKIR, S., BIGHAM, M., RICHTER, A., FEI, Z. & JANDER, G. 2019a. Genome sequence of the corn leaf aphid (*Rhopalosiphum maidis* Fitch). *GigaScience,* 8.

CHEN, W., WOSULA, E. N., HASEGAWA, D. K., CASINGA, C., SHIRIMA, R. R., FIABOE, K. K. M., HANNA, R., FOSTO, A., GOERGEN, G., TAMO, M., MAHUKU, G., MURITHI, H. M., TRIPATHI, L., MWARE, B., KUMAR, L. P., NTAWURUHUNGA, P., MOYO, C., YOMENI, M., BOAHEN, S., EDET, M., AWOYALE, W., WINTERMANTEL, W. M., LING, K. S., LEGG, J. P. & FEI, Z. 2019b. Genome of the African cassava whitefly Bemisia tabaci and distribution and genetic diversity of cassava-colonizing whiteflies in Africa. *Insect Biochem Mol Biol,* 110**,** 112-120.

ETTINGER, C. L., BYRNE, F. J., COLLIN, M. A., CARTER-HOUSE, D., WALLING, L. L., ATKINSON, P. W., REDAK, R. A. & STAJICH, J. E. 2021. Improved draft reference genome for the Glassy-winged Sharpshooter (Homalodisca vitripennis), a vector for Pierce's disease. *G3-Genes Genomes Genetics,* 11.

GIORDANO, R., DONTHU, R. K., ZIMIN, A. V., JULCA CHAVEZ, I. C., GABALDON, T., VAN MUNSTER, M., HON, L., HALL, R., BADGER, J. H., NGUYEN, M., FLORES, A., POTTER, B., GIRAY, T., SOTO-ADAMES, F. N., WEBER, E., MARCELINO, J. A. P., FIELDS, C. J., VOEGTLIN, D. J., HILL, C. B., HARTMAN, G. L. & SOYBEAN APHID RESEARCH, C. 2020. Soybean aphid biotype 1 genome: Insights into the invasive biology and adaptive evolution of a major agricultural pest. *Insect Biochem Mol Biol,* 120**,** 103334.

HUANG, H.-J., YE, Y.-X., YE, Z.-X., YAN, X.-T., WANG, X., WEI, Z.-Y., CHEN, J.-P., LI, J.-M., SUN, Z.-T. & ZHANG, C.-X. 2021. Chromosome-level genome assembly of the bean bug *Riptortus pedestris*. *Molecular Ecology Resources,* 21**,** 2423-2436.

JIANG, X., ZHANG, Q., QIN, Y., YIN, H., ZHANG, S., LI, Q., ZHANG, Y., FAN, J. & CHEN, J. 2019. A chromosome-level draft genome of the grain aphid *Sitobion miscanthi*. *GigaScience,* 8.

KORGAONKAR, A., HAN, C., LEMIRE, A. L., SIWANOWICZ, I., BENNOUNA, D., KOPEC, R. E., ANDOLFATTO, P., SHIGENOBU, S. & STERN, D. L. 2021. A novel family of secreted insect proteins linked to plant gall development. *Curr Biol,* 31**,** 1836-1849 e12.

LI, Y., PARK, H., SMITH, T. E. & MORAN, N. A. 2019. Gene Family Evolution in the Pea Aphid Based on Chromosome-Level Genome Assembly. *Molecular Biology and Evolution,* 36**,** 2143-2156.

MA, W., XU, L., HUA, H., CHEN, M., GUO, M., HE, K., ZHAO, J. & LI, F. 2021. Chromosomal-level genomes of three rice planthoppers provide new insights into sex chromosome evolution. *Mol Ecol Resour,* 21**,** 226-237.

MATHERS, T. C., CHEN, Y., KAITHAKOTTIL, G., LEGEAI, F., MUGFORD, S. T., BAA-PUYOULET, P., BRETAUDEAU, A., CLAVIJO, B., COLELLA, S., COLLIN, O., DALMAY, T., DERRIEN, T., FENG, H., GABALDON, T., JORDAN, A., JULCA, I., KETTLES, G. J., KOWITWANICH, K., LAVENIER, D., LENZI, P., LOPEZ-GOMOLLON, S., LOSKA, D., MAPLESON, D., MAUMUS, F., MOXON, S., PRICE, D. R., SUGIO, A., VAN MUNSTER, M., UZEST, M., WAITE, D., JANDER, G., TAGU, D., WILSON, A. C., VAN OOSTERHOUT, C., SWARBRECK, D. & HOGENHOUT, S. A. 2017. Rapid transcriptional plasticity of duplicated gene clusters enables a clonally reproducing aphid to colonise diverse plant species. *Genome Biol,* 18**,** 27.

MATHERS, T. C., MUGFORD, S. T., HOGENHOUT, S. A. & TRIPATHI, L. 2020. Genome Sequence of the Banana Aphid, Pentalonia nigronervosa Coquerel (Hemiptera: Aphididae) and Its Symbionts. *G3 (Bethesda),* 10**,** 4315-4321.

NICHOLSON, S. J., NICKERSON, M. L., DEAN, M., SONG, Y., HOYT, P. R., RHEE, H., KIM, C. & PUTERKA, G. J. 2015. The genome of Diuraphis noxia, a global aphid pest of small grains. *BMC Genomics,* 16**,** 429.

QUAN, Q., HU, X., PAN, B., ZENG, B., WU, N., FANG, G., CAO, Y., CHEN, X., LI, X., HUANG, Y. & ZHAN, S. 2019. Draft genome of the cotton aphid Aphis gossypii. *Insect Biochem Mol Biol,* 105**,** 25-32.

RODRIGUES, A. S., SILVA, S. E., PINA-MARTINS, F., LOUREIRO, J., CASTRO, M., GHARBI, K., JOHNSON, K. P., DIETRICH, C. H., BORGES, P. A., QUARTAU, J. A., JIGGINS, C. D., PAULO, O. S. & SEABRA, S. G. 2016. Assessing genotype-phenotype associations in three dorsal colour morphs in the meadow spittlebug Philaenus spumarius (L.) (Hemiptera: Aphrophoridae) using genomic and transcriptomic resources. *BMC Genet,* 17**,** 144.

ROSENFELD, J. A., REEVES, D., BRUGLER, M. R., NARECHANIA, A., SIMON, S., DURRETT, R., FOOX, J., SHIANNA, K., SCHATZ, M. C., GANDARA, J., AFSHINNEKOO, E., LAM, E. T., HASTIE, A. R., CHAN, S., CAO, H., SAGHBINI, M., KENTSIS, A., PLANET, P. J., KHOLODOVYCH, V., TESSLER, M., BAKER, R., DESALLE, R., SORKIN, L. N., KOLOKOTRONIS, S. O., SIDDALL, M. E., AMATO, G. & MASON, C. E. 2016. Genome assembly and geospatial phylogenomics of the bed bug Cimex lectularius. *Nat Commun,* 7**,** 10164.

SPARKS, M. E., BANSAL, R., BENOIT, J. B., BLACKBURN, M. B., CHAO, H., CHEN, M., CHENG, S., CHILDERS, C., DINH, H., DODDAPANENI, H. V., DUGAN, S., ELPIDINA, E. N., FARROW, D. W., FRIEDRICH, M., GIBBS, R. A., HALL, B., HAN, Y., HARDY, R. W., HOLMES, C. J., HUGHES, D. S. T., IOANNIDIS, P., CHEATLE JARVELA, A. M., JOHNSTON, J. S., JONES, J. W., KRONMILLER, B. A., KUNG, F., LEE, S. L., MARTYNOV, A. G., MASTERSON, P., MAUMUS, F., MUNOZ-TORRES, M., MURALI, S. C., MURPHY, T. D., MUZNY, D. M., NELSON, D. R., OPPERT, B., PANFILIO, K. A., PAULA, D. P., PICK, L., POELCHAU, M. F., QU, J., REDING, K., RHOADES, J. H., RHODES, A., RICHARDS, S., RICHTER, R., ROBERTSON, H. M., ROSENDALE, A. J., TU, Z. J., VELAMURI, A. S., WATERHOUSE, R. M., WEIRAUCH, M. T., WELLS, J. T., WERREN, J. H., WORLEY, K. C., ZDOBNOV, E. M. & GUNDERSEN-RINDAL, D. E. 2020. Brown marmorated stink bug, Halyomorpha halys (Stal), genome: putative underpinnings of polyphagy, insecticide resistance potential and biology of a top worldwide pest. *BMC Genomics,* 21**,** 227.

TIWARI, J. K., MANDADI, N., SRIDHAR, J., MANDAL, V., GHOSH, A., KARDILE, H. B., NAGA, K. C., SHAH, M. A., RAWAT, S., VENKATESWARLU, V., MALIK, K., BHATNAGAR, A., CHAKRABARTI, S. K., KUMAR, M., RAO, A. R. & RAI, A. 2021. Draft genome sequencing of the foxglove aphid (Aulacorthum solani Kaltenbach), a vector of potato viruses, provides insights on virulence genes. *Journal of Asia-Pacific Entomology,* 24**,** 93-102.

WHITE, H. B. & PIRRO, S. 2021. The complete genome sequences of two species of seventeen-year cicadas: Magicicada septendecim and Magicicada septendecula. *F1000Research,* 10.

XIE, W., CHEN, C., YANG, Z., GUO, L., YANG, X., WANG, D., CHEN, M., HUANG, J., WEN, Y., ZENG, Y., LIU, Y., XIA, J., TIAN, L., CUI, H., WU, Q., WANG, S., XU, B., LI, X., TAN, X., GHANIM, M., QIU, B., PAN, H., CHU, D., DELATTE, H., MARUTHI, M. N., GE, F., ZHOU, X., WANG, X., WAN, F., DU, Y., LUO, C., YAN, F., PREISSER, E. L., JIAO, X., COATES, B. S., ZHAO, J., GAO, Q., XIA, J., YIN, Y., LIU, Y., BROWN, J. K., ZHOU, X. J. & ZHANG, Y. 2017. Genome sequencing of the sweetpotato whitefly Bemisia tabaci MED/Q. *Gigascience,* 6**,** 1-7.

YANG, P., YU, S., HAO, J., LIU, W., ZHAO, Z., ZHU, Z., SUN, T., WANG, X. & SONG, Q. 2019. Genome sequence of the Chinese white wax scale insect Ericerus pela: the first draft genome for the Coccidae family of scale insects. *Gigascience,* 8.

YE, Y. X., ZHANG, H. H., LI, D. T., ZHUO, J. C., SHEN, Y., HU, Q. L. & ZHANG, C. X. 2021. Chromosome-level assembly of the brown planthopper genome with a characterized Y chromosome. *Mol Ecol Resour,* 21**,** 1287-1298.

ZHANG, S., GAO, X., WANG, L., JIANG, W., SU, H., JING, T., CUI, J., ZHANG, L. & YANG, Y. 2022. Chromosome-level genome assemblies of two cotton-melon aphid *Aphis gossypii* biotypes unveil mechanisms of host adaption. *Mol Ecol Resour,* 22**,** 1120-1134.

ZHU, B., WEI, R., HUA, W., LI, L., ZHANG, W., LIANG, P. & GAO, X. 2022. A High-Quality Chromosome-Level Assembly Genome Provides Insights into Wing Dimorphism and Xenobiotic Detoxification in Metopolophium Dirhodum (Walker). Research Square.

ZUCCHI, M. I., CORDEIRO, E. M. G., ALLEN, C., NOVELLO, M., VIANA, J. P. G., BROWN, P. J., MANJUNATHA, S., OMOTO, C., PINHEIRO, J. B. & CLOUGH, S. J. 2019. Patterns of Genome-Wide Variation, Population Differentiation and SNP Discovery of the Red Banded Stink Bug (Piezodorus guildinii). *Sci Rep,* 9**,** 14480.
