# Supplementary material for: Gene Editing and Genetic Control of Hemipteran Pests: Progress, Challenges and Perspectives
Source: Front Bioeng Biotechnol. 2022 Jun 7;10:900785. doi: 10.3389/fbioe.2022.900785 (PMC9209771; doi:10.3389/fbioe.2022.900785)
Supplement: Supplementary file 2 [file Table2.docx]

**Supplementary Table S2:** **Cell culture lines of Hemiptera.**

| **Cell Culture Line** | **Species** | **Tissue of Origin** | **Reference** |
| --- | --- | --- | --- |
| - | *AgatIia constricta*  (Constricted leafhopper) | embryo | Chiu and Black, 1967 |
| - | *Agallia quadripunctata*  (Four-spotted clover leafhopper) | embryo | Chiu and Black, 1967 |
| - | *Aceratagallia sanguinolenta*  (Clover Leafhopper) | embryo | Chiu and Black, 1967 |
| - | *Agalliopsis novella*  (Agalliopsis) | embryo | Chiu and Black, 1967 |
| BCIRL-AtE-CLG11, BCIRL-AtE-CLG15 | *Anasa tristis*  (Squash bug) | embryo | Goodman et al., 2017 |
| Btb(Ba)97 | *Bemisia tabaci* (B biotype)  *(Whitefly)* | embryo | Hunter and Polston, 2001 |
| - | *Cacopsylla melanoneura* | immunocytes | Monti et al. 2014 |
| - | *Cacopsylla pyri*  (Pear psyllid) | immunocytes | Monti et al. 2014 |
| - | *Cacopsylla crataegi* | immunocytes | Monti et al. 2014 |
| CT 1 | *Circulifer tenellusto*  (Beet leafhopper) | embryo | Wayadande and Fletcher, 1998 |
| NIVI-CH-440, NIVI-CH-442, NIVI-CH-445 | *Cimex hemipterus*  (Tropical bed bug) | embryo | Pant et al., 1988 |
| - | *Colladonus montanus*  (Mountain Leafhopper) | Not stated | Richardson and Jensen, 1971 |
| DcHH-1 | *Diaphorina citri*  (Asian citrus psillyd) | embryo | Marutani-Hert et al., 2009 |
| GWSS-Z15, GWSS-G3, GWSS-LH | *Homalodisca vitripennis*  (Glassy-winged sharpshooter) | embryo | Kamita et al., 2005 |
| BPH | *Nilaparvata lugens*  (Brown planthopper) | embryo | Xu et al., 2014 |
| - | *Nephotettix cincticeps*  (Rice green leafhopper) | embryo | Mitsuhashi and Kono, 1975 |
| TI- 32 | *Triatoma infestans*  (kissing bug) | embryo | Pudney and Lanar, 1977 |
| - | *Myzus persicae*  (green peach aphid) | embryo/ovaries | Adam and Sander, 1976 |
| - | *Hyperomyzus Zactucae*  (Blackcurrant-sowthistle aphid) | embryo/ovaries | Peters and Black, 1970 |
| - | *Sogatella furcifera*  (white-backed planthopper) | embryo | Jia et al., 2012 |

**References**

ADAM, G. & SANDER, E. 1976. Isolation and Culture of Aphid Cells for Assay of Insect-Transmitted Plant Viruses. *Virology,* 70**,** 502-508.

CHIU, R. J. & BLACK, L. M. 1967. Monolayer Cultures of Insect Cell Lines and Their Inoculation with a Plant Virus. *Nature,* 215**,** 1076.

GOODMAN, C. L., LINCOLN, T. R., LI, Y. F., RINGBAUER, J., ZHOU, K. L. & STANLEY, D. 2017. Eicosanoid Inhibitors Impact Protein Expression in an Insect Cell Line. *In Vitro Cellular & Developmental Biology-Animal,* 53**,** S43-S43.

HUNTER, W. B. & POLSTON, J. E. 2001. Development of a continuous whitefly cell line [Homoptera: Aleryrodidae: Bemisia tabaci (Gennadius)] for the study of begomovirus. *J Invertebr Pathol,* 77**,** 33-6.

JIA, D. S., CHEN, H. Y., ZHENG, A. L., CHEN, Q., LIU, Q. F., XIE, L. H., WU, Z. J. & WEI, T. Y. 2012. Development of an Insect Vector Cell Culture and RNA Interference System To Investigate the Functional Role of Fijivirus Replication Protein. *Journal of Virology,* 86**,** 5800-5807.

KAMITA, S. G., DO, Z. N., SAMRA, A. I., HAGLER, J. R. & HAMMOCK, B. D. 2005. Characterization of cell lines developed from the glassy-winged sharpshooter, Homalodisca coagulata (Hemiptera : Cicadellidae). *In Vitro Cellular & Developmental Biology-Animal,* 41**,** 149-153.

MARUTANI-HERT, M., HUNTER, W. B., KATSAR, C. S., SINISTERRA, X. H., HALL, D. G. & POWELL, C. A. 2009. Reovirus-Like Sequences Isolated from Adult Asian Citrus Psyllid, (Hemiptera: Psyllidae: Diaphorina Citri). *Florida Entomologist,* 92**,** 314-320.

MITSUHASHI, J. & KONO, Y. 1975. Intracellular microorganisms in the green rice leafhopper, Nephotettix cincticeps UHLER (Hemiptera: Deltocephalidae). *Applied Entomology and Zoology,* 10**,** 1-9.

MONTI, M., MANDRIOLI, M., BEXTINE, B., HUNTER, W. B., ALMA, A. & TEDESCHI, R. 2014. Maintenance of primary cell cultures of immunocytes from Cacopsylla spp. psyllids: a new in vitro tool for the study of crop pest insects. *In Vitro Cellular & Developmental Biology-Animal,* 50**,** 797-801.

PANT, U., DHANDA, V., ARAGADE, S. P. & BANERJEE, K. 1988. Establishment of 3 Cell-Lines from the Embryonic Tissue of Cimex-Hemipterus (F) (Hemiptera, Cimicidae). *In Vitro Cellular & Developmental Biology,* 24**,** 1201-1203.

PETERS, D. & BLACK, L. M. 1970. Infection of Primary Cultures of Aphid Cells with a Plant Virus. *Virology,* 40**,** 847-&.

PUDNEY, M. & LANAR, D. 1977. Establishment and Characterization of a Cell Line (Btc-32) from Triatomine Bug, Triatoma-Infestans (Klug) (Hemiptera-Reduviidae). *Annals of Tropical Medicine and Parasitology,* 71**,** 109-118.

RICHARDSON, J. & JENSEN, D. D. 1971. Tissue Culture of Monolayer Cell Lines of Colladonus-Montanus (Homoptera-Cicadellidae) a Vector of Causal Agent of Western X-Disease of Peach. *Annals of the Entomological Society of America,* 64**,** 722-+.

WAYADANDE, A. C. & FLETCHER, J. 1998. Development and use of an established cell line of the leafhopper Circulifer tenellus to characterize Spiroplasma citri - Vector interactions. *Journal of Invertebrate Pathology,* 72**,** 126-131.

XU, Y. P., CHEN, Y. H. & YU, X. P. 2014. Cell culture of the rice brown planthopper, Nilaparvata lugens StAyenl (Hemiptera: Delphacidae). *In Vitro* *Cellular & Developmental Biology-Animal,* 50**,** 384-388
